# Neoplastic fibrocytes play an essential role in bone marrow fibrosis in Jak2V617F-induced primary myelofibrosis mice

**DOI:** 10.1038/s41375-020-0880-3

**Published:** 2020-05-29

**Authors:** Yoshinori Ozono, Kotaro Shide, Takuro Kameda, Ayako Kamiunten, Yuki Tahira, Masaaki Sekine, Keiichi Akizuki, Kenichi Nakamura, Hisayoshi Iwakiri, Mitsue Sueta, Tomonori Hidaka, Yoko Kubuki, Shojiro Yamamoto, Satoru Hasuike, Akira Sawaguchi, Kenji Nagata, Kazuya Shimoda

**Affiliations:** 1 Department of Gastroenterology and Hematology, Faculty of Medicine, University of Miyazaki, Miyazaki, Japan; 2 Department of Anatomy, Ultrastructural Cell Biology, Faculty of Medicine, University of Miyazaki, Miyazaki, Japan

**Keywords:** Cancer models, Bone marrow transplantation

## Abstract

Primary myelofibrosis (PMF) is a myeloproliferative neoplasm (MPN) characterized by clonal myeloproliferation, progressive bone marrow (BM) fibrosis, splenomegaly, and anemia. BM fibrosis was previously thought to be a reactive phenomenon induced by mesenchymal stromal cells that are stimulated by the overproduction of cytokines such as transforming growth factor (TGF)-β1. However, the involvement of neoplastic fibrocytes in BM fibrosis was recently reported. In this study, we showed that the vast majority of collagen- and fibronectin-producing cells in the BM and spleens of Jak2V617F-induced myelofibrosis (MF) mice were fibrocytes derived from neoplastic hematopoietic cells. Neoplastic monocyte depletion eliminated collagen- and fibronectin-producing fibrocytes in BM and spleen, and ameliorated most characteristic MF features in Jak2V617F transgenic mice, including BM fibrosis, anemia, and splenomegaly, while had little effect on the elevated numbers of megakaryocytes and stem cells in BM, and leukothrombocytosis in peripheral blood. TGF-β1, which was produced by hematopoietic cells including fibrocytes, promoted the differentiation of neoplastic monocytes to fibrocytes, and elevated plasma TGF-β1 levels were normalized by monocyte depletion. Collectively, our data suggest that neoplastic fibrocytes are the major contributor to BM fibrosis in PMF, and TGF-β1 is required for their differentiation.

## Introduction

Primary myelofibrosis (PMF) is a myeloproliferative neoplasm (MPN) characterized by an increased number of atypical megakaryocytes, decreased erythropoiesis, and progressive fibrosis in bone marrow (BM) [1, 2]. Constitutive activation of JAK–STAT signaling cascades, caused for example by JAK2V617F, are thought to play an essential role in MPN pathology [3, 4]. The JAK1/2 kinase inhibitor ruxolitinib ameliorates PMF-associated symptoms and splenomegaly, but rarely resolves BM fibrosis [5, 6].

BM fibrosis in PMF has been thought to be a reactive phenomenon caused by the overproduction of cytokines such as transforming growth factor (TGF)-β1, mainly by megakaryocytes and platelets [7–9]. Such cytokines stimulate wild-type (WT) mesenchymal stromal cells (MSCs) to produce collagen and fibronectin and to induce BM fibrosis. However, Verstovsek et al. reported that BM cells (BMCs) from PMF patients harbored an abundance of clonal, neoplastic fibrocytes that produced collagen and fibronectin and that contributed to BM fibrosis in PMF [10]. In addition, Maekawa et al. reported that myeloproliferative leukemia protein activation directly induced fibrocyte differentiation in murine cell lines [11]. In this study, we aimed to evaluate the role of neoplastic fibrocytes in the pathogenesis of BM fibrosis induced by Jak2V617F.

## Materials and methods

Jak2V617F transgenic (TG) mice were generated as previously described [12]. CD11b-diphtheria toxin receptor (DTR) TG mice were purchased from The Jackson Laboratory (Bar Harbor, ME, USA) [13]. These mice were crossed to generate Jak2V617F/CD11b-DTR compound TG. To deplete monocytes in vivo, recipient mice transplanted with BMCs from CD11b-DTR TG mice or Jak2V617F/CD11b-DTR TG mice were administered 15 ng/g diphtheria toxin (DT) (List Biological Labs, Campbell, CA, USA) by intraperitoneal injection every 2 days for 8 weeks. All experiments were approved by the Animal Experiment Committee of the University of Miyazaki. The detailed methods are described in the supplementary methods and supplementary tables (Supplementary Tables 1 and 2).

## Results

### BMCs from Jak2V617F TG contain many fibrocytes

**Fig. 1:**
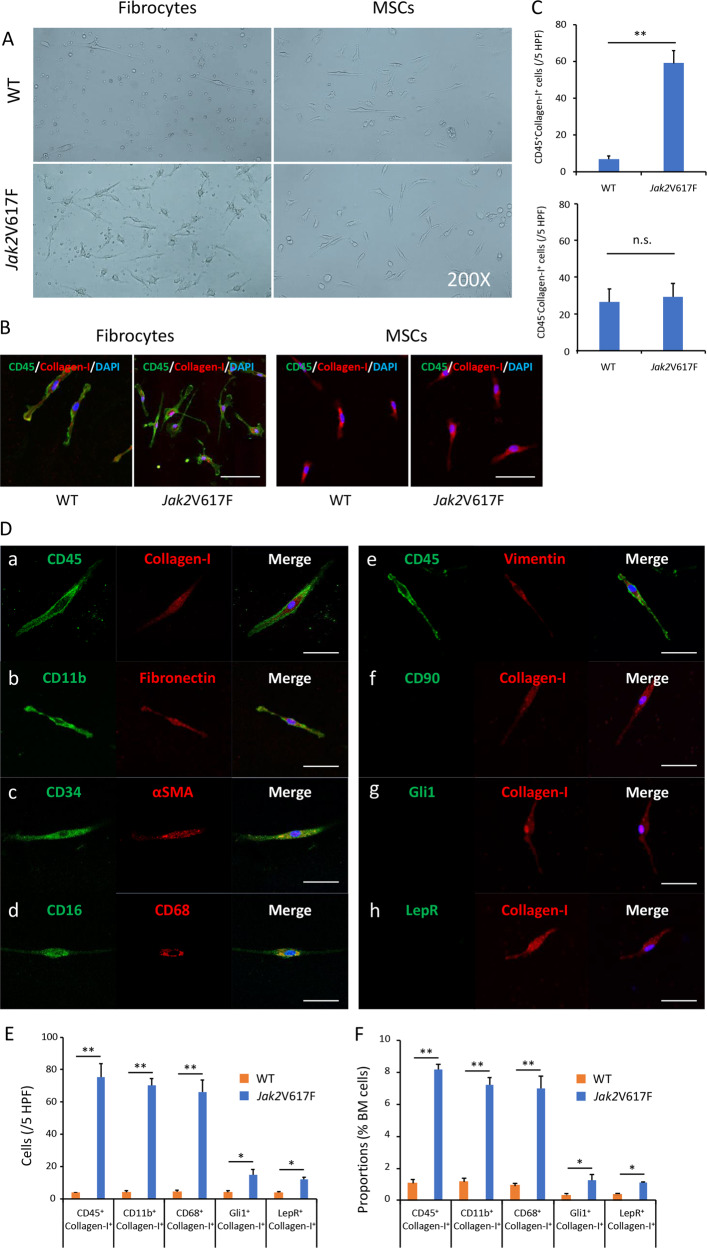
Morphology of fibrocytes derived from BM of Jak2V617F and WT mice. **a** Phase-contrast micrographs of cultured BM MNCs from WT (top) and Jak2V617F (bottom) mice in conditions that promote differentiation of monocytes to fibrocytes (left) or that support MSCs proliferation (right). Representative images are presented from 24 wells of cultured BM MNCs from three mice of each type. **b** Spindle-shaped cells in BM MNCs that were cultured in conditions that promote differentiation to fibrocytes were CD45^+^Collagen-I^+^, whereas spindle-shaped cells in BM MNCs that were cultured in conditions that support MSC proliferation are CD45^−^Collagen-I^+^ by immunofluorescence imaging. Bars: 50 μm. **c** The number of CD45^+^Collagen-I^+^ or CD45^−^Collagen-I^+^ cells per five random high-powered fields (HPFs) in BM MNCs from WT and Jak2V617F TG mice (*n* = 3 in each group) that were cultured in conditions that promote differentiation to fibrocytes or that support MSCs proliferation, respectively. **d** Representative immunofluorescence imaging of spindle-shaped cells cultured from BM MNCs of Jak2V617F mice in conditions that promote differentiation of monocytes to fibrocytes. DAPI staining is shown in blue (merge). Bars: 50 μm. (a) Costaining for CD45 and Collagen-I. (b) Costaining for CD11b and fibronectin. (c) Costaining for CD34 and αSMA. (d) Costaining for CD16 and CD68. (e) Costaining for CD45 and vimentin. (f) Costaining for CD90 and Collagen-I. (g) Costaining for Gli1 and Collagen-I. (h) Costaining for LepR and Collagen-I. **e** The number of CD45^+^ Collagen-I^+^, CD11b^+^ Collagen-I^+^, and CD68^+^ Collagen-I^+^ fibrocytes and Gli1^+^Collagen-I^+^ and LepR^+^Collagen-I^+^ myofibroblasts in five random HPFs from BM of WT mice (*n* = 3) and Jak2V617F TG mice (*n* = 3) by immunofluorescence analysis. **f** The proportion of CD45^+^ Collagen-I^+^, CD11b^+^Collagen-I^+^, and CD68^+^Collagen-I^+^fibrocytes and Gli1^+^Collagen-I^+^ and LepR^+^Collagen-I^+^ myofibroblasts in BMCs of WT and Jak2V617F TG mice by FACS analysis (*n* = 5 in each group). Data are expressed as means ± SEM. The two-tailed student's *t* test was used (**c**, **e**, **f**). **P < 0.01, *P < 0.05. n.s. not significant. The representative result is shown in three independent experiments (**a**, **b**, **d**).

BM mononuclear cells (MNCs) from Jak2V617F TG or WT mice were cultured on 24-well dishes in conditions that promoted differentiation of monocytes to fibrocytes or those that supported MSCs proliferation (Fig. 1a). After 5 days, a large number of long spindle-shaped cells, together with small numbers of round cells, were observed in dishes with Jak2V617F BM MNCs in conditions that promoted differentiation of monocytes to fibrocytes under phase-contrast microscopy. The spindle-shaped cells were also observed in dishes cultured with WT BM MNCs, but were far fewer in number than in dishes cultured with Jak2V617F BM MNCs (Fig. 1a–c). These long spindle-shaped cells were positive for CD45 and Collagen-I. We next evaluated the characteristics of these cells using immunofluorescence. As shown in Fig. 1d, the cells produced collagen and fibronectin, and were positive for hematopoietic markers such as CD45, CD11b, CD34, CD16, and CD68, as well as extracellular matrix proteins such as alpha smooth muscle actin (αSMA) and vimentin; however, they were negative for MSCs markers such as CD90, glioma-associated oncogene homolog 1 (Gli1), and leptin receptor (LepR), indicating that they were fibrocytes. The number of CD45^−^Collagen-I^+^ MSCs was the same in WT and Jak2V617F TG mice (Fig. 1a–c).

We next performed histological and fluorescence-activated cell sorter (FACS) analysis of BM from Jak2V617F TG mice. These mice developed myelofibrosis (MF), and an immunofluorescence analysis showed an overabundance of collagen- and fibronectin-producing cells in BM (Supplementary Figs. 1–4 and Fig. 1e). About 8% of BMCs from Jak2V617F TG were collagen-producing cells, compared with fewer than 1% in WT mice (Fig. 1f). Most collagen-producing BMCs from Jak2V617F TG mice coexpressed hematopoietic markers such as CD45, CD11b, and CD68, indicating that these cells were fibrocytes. Gli1^+^Collagen-I^+^ and LepR^+^Collagen-I^+^ myofibroblasts each comprised 1–2% of BMCs from Jak2V617F TG mice, which was still a higher percentage than that in WT mice.

### Most collagen- and fibronectin-producing cells in the BM and spleens of Jak2V617F-induced MF mice are neoplastic fibrocytes

We next performed histological analysis of BM from recipient mice transplanted with Jak2V617F fetal liver cells (FLCs). As we previously reported, recipient mice transplanted with Jak2V617F FLCs developed MF, but not mice transplanted with WT FLCs (Supplementary Fig. 5) [14]. Immunofluorescence analysis showed an overabundance of collagen-producing cells in BM from mice transplanted with Jak2V617F FLCs, whereas they were scarcely observed in BM from mice transplanted with WT FLCs. Many collagen-producing cells coexpressed CD45.2, CD11b, and CD68, and a few expressed Gli1 and LepR, indicating that BM from mice transplanted with Jak2V617F FLCs contained many fibrocytes and a few myofibroblasts.

**Fig. 2:**
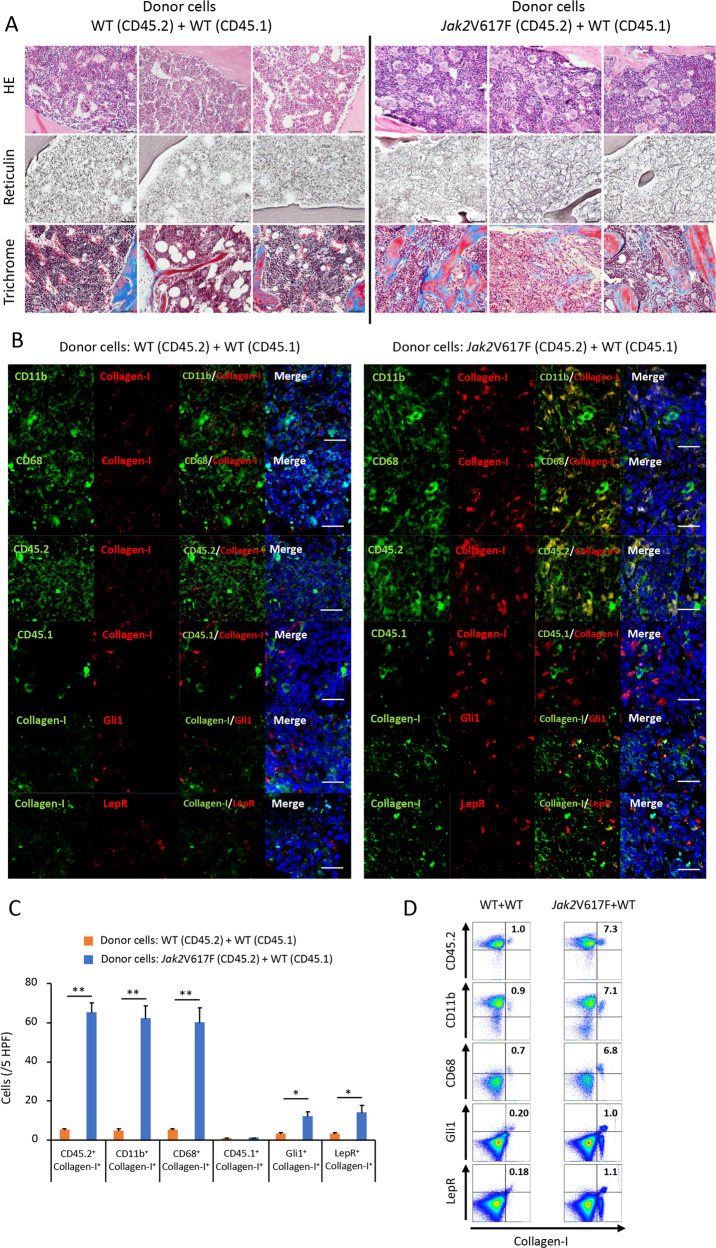
Increased numbers of neoplastic fibrocytes in BM from recipient mice transplanted with a mixture of Jak2V617F BMCs and WT BMCs. **a** Hematoxylin and eosin (HE), reticulin silver, and Masson trichrome staining of BM sections from recipient mice transplanted with a mixture of Jak2V617F BMCs and WT BMCs in a 5:1 ratio (*n* = 3), and those transplanted with a mixture of WT BMCs and WT BMCs (*n* = 3). Images from all six mice are presented. Bars: 50 μm. **b** Immunofluorescence imaging of BM sections from recipient mice (CD45.1) transplanted with the mixture of Jak2V617F BMCs (CD45.2) plus WT BMCs (CD45.1) (right) and recipient mice (CD45.1) transplanted with the mixture of WT BMCs (CD45.2) plus WT BMCs (CD45.1) (left). DAPI staining is shown in blue (merge). Bars: 20 μm. The representative result is shown in three independent experiments. **c** The numbers of cells with the following characteristics: neoplastic fibrocytes positive for CD45.2/Collagen-I, CD11b/Collagen-I, and CD68/Collagen-I; WT fibrocytes positive for CD45.1/Collagen-I; and myofibroblasts positive for Gli1/Collagen-I and LepR/Collagen-I. Cell numbers are presented as the average of five random HPFs from three mice of each type. Data are expressed as means ± SEM. The two-tailed student’s *t* test was used. **P < 0.01. *P < 0.05. **d** Representative plots of FACS analysis of CD45.2^+^Collagen-I^+^, CD11b^+^Collagen-I^+^, and CD68^+^Collagen-I^+^fibrocytes, and Gli1^+^Collagen-I^+^ and LepR^+^Collagen-I^+^ myofibroblasts in BMCs isolated from recipient mice (CD45.1) transplanted with the mixture of Jak2V617F BMCs (CD45.2) plus WT BMCs (CD45.1) and recipient mice (CD45.1) transplanted with the mixture of WT BMCs (CD45.2) plus WT BMCs (CD45.1). The reproducibility was confirmed by five experiments.

In BM of MPN patients, there are two types of hematopoietic cells: WT hematopoietic cells and neoplastic hematopoietic cells. To distinguish the effect of each cell type on BM fibrosis, we transplanted BMCs either from WT or Jak2V617F TG mice (CD45.2), together with competitor WT BMCs (CD45.1) in a 5:1 ratio, into irradiated WT recipient mice (CD45.1). Recipient mice transplanted with the mixture of Jak2V617F BMCs and WT BMCs developed MF (Fig. 2a).

Immunofluorescence analysis of BM from recipient mice transplanted with Jak2V617F BMCs and WT BMCs showed an overabundance of cells expressing collagen and fibronectin, and also CD11b and CD68 (Fig. 2b and Supplementary Fig. 6A), and they were positive for CD45.2 (a marker for Jak2V617F hematopoietic cells) but not CD45.1 (a marker for WT hematopoietic cells). There was a smaller number of cells coexpressing Gli1 or LepR with Collagen-I or fibronectin. These results indicated that most of the collagen- and fibronectin-producing cells in BM of Jak2V617F-induced MF mice were fibrocytes derived from neoplastic hematopoietic cells, while a few of them were myofibroblasts that originated from WT MSCs. In BM from recipient mice transplanted with WT BMCs (CD45.2) and competitor WT BMCs (CD45.1), the number of cells that produced collagen and fibronectin was very small. A few of the collagen-producing cells were positive for Gli1 and LepR, indicating that they were myofibroblasts, but their number was much smaller than in recipient mice transplanted with Jak2V617F BMCs and competitor WT BMCs (Fig. 2c). In FACS analysis, about 7% of BMCs from recipient mice transplanted with Jak2V617F cells were collagen-producing cells that coexpressed hematopoietic markers such as CD11b and CD68, compared with about 1% in recipient mice transplanted with WT cells (Fig. 2d and Supplementary Fig. 6B). Most collagen-producing cells from recipient mice transplanted with Jak2V617F cells coexpressed CD45.2, indicating that these cells were fibrocytes derived from neoplastic hematopoietic cells. Collagen-producing cells expressing Gli1 and LepR (myofibroblasts) was 1% in BM from recipient mice transplanted with Jak2V617F cells, which was still a higher percentage than that in BM from recipient mice transplanted with WT cells.

### Neoplastic monocyte depletion drastically decreases the number of collagen- and fibronectin-producing fibrocytes in BM and spleen, and ameliorates BM fibrosis, anemia, and splenomegaly in Jak2V617F-induced MF mice

**Fig. 3:**
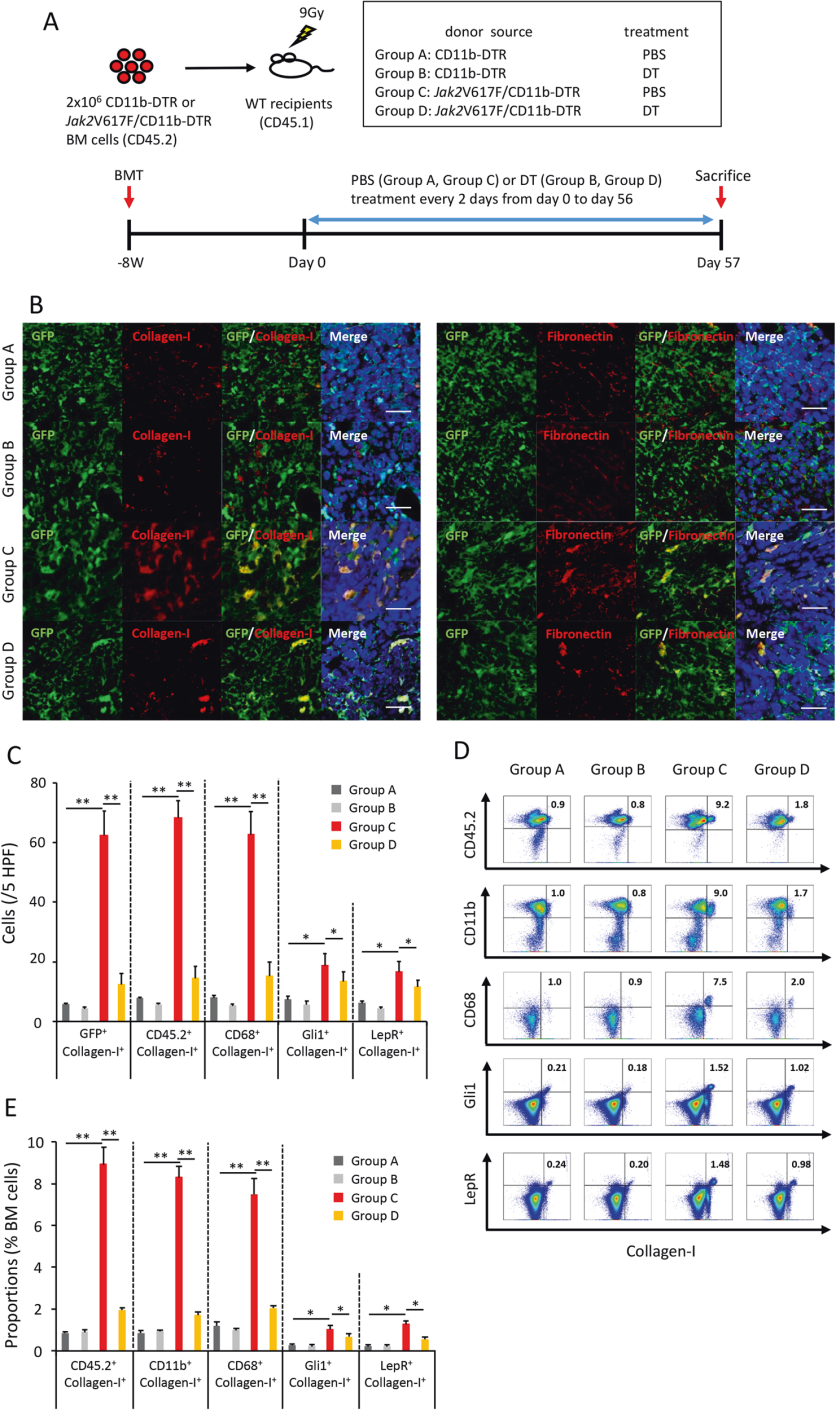
Monocyte depletion reduces the number of neoplastic fibrocytes in BM from recipient mice transplanted with Jak2V617F BMCs. **a** Outline of experimental design. BMCs from CD11b-DTR TG (groups A, B) or Jak2V617F/CD11b-DTR TG (groups C, D) were transplanted into lethally irradiated recipient mice (*n* = 12 in group A and C, *n* = 14 in group B and D). GFP was expressed under the CD11b promoter. After 8 weeks, treatment with PBS (groups A, C) or DT (groups B, D) was performed every 2 days for 8 weeks. **b** Immunofluorescence imaging of BM sections from each group of recipient mice (*n* = 3 in each group). DAPI staining is shown in blue (merge). Bars: 20 μm. The representative result is shown in three independent experiments. **c** The number of GFP^+^Collagen-I^+^, CD45.2^+^Collagen-I^+^, and CD68^+^Collagen-I^+^fibrocytes, and Gli1^+^Collagen-I^+^ and LepR^+^Collagen-I^+^ myofibroblasts in BM. Cell numbers are presented as the average of five random HPFs (*n* = 3 in each group). **d** Representative plots of FACS analysis of collagen-producing cells (same as Fig. 2d) in BMCs from each recipient mouse group after 8-week PBS or DT treatment. The reproducibility was confirmed by six experiments. **e** The proportion of fibrocytes and myofibroblasts in recipient mice transplanted with CD11b-DTR BMCs or Jak2V617F/CD11b-DTR BMCs (*n* = 6 in group A and B, *n* = 8 in group C and D). Data are expressed as means ± SEM. One-way ANOVA followed by the Tukey–Kramer test was used (**c**, **e**). **P < 0.01, *P < 0.05.

We next aimed to deplete neoplastic fibrocytes and observe the effects on BM fibrosis. Fibrocytes were differentiated from monocytes. To deplete monocytes in vivo, we adopted TG with restricted expression of human DTR under the control of the CD11b promoter, in which circulating monocytes could be selectively ablated after DT administration [15]. However, due to the expression of DTR on nonhematopoietic cells, repeated DT injection in CD11b-DTR mice is lethal or results in extrahematopoietic toxicity [16]. In order to ablate transgene lineages for longer periods without adverse effects, we mated Jak2V617F TG mice with CD11b-DTR TG mice, and obtained Jak2V617F/CD11b-DTR compound mice. We then transplanted CD11b-DTR or Jak2V617F/CD11b-DTR BMCs into irradiated WT recipient mice (Fig. 3a). One day after intraperitoneal DT administration, the number of CD11b^+^F4/80^+^ monocytes decreased drastically both in recipient mice transplanted with CD11b-DTR BMCs and in those transplanted with Jak2V617F/CD11b-DTR BMCs (Supplementary Fig. 7). Monocyte depletion in both types of mice was maintained for 8 weeks by every-other-day DT administration. DT treatment had little effect on Mac1^+^Gr1^+^, CD3^+^, or B220^+^ cells.

Green fluorescent protein (GFP) was expressed under the CD11b promoter in CD11b-DTR TG mice, and GFP signaling in cells expressing CD11b was detected under fluorescent microscopy. Compared with CD11b-DTR mice treated with PBS (group A), BM from Jak2V617F/CD11b-DTR mice treated with PBS (group C) contained many more collagen- and fibronectin-producing cells and were positive for GFP, indicating a hematopoietic origin with CD11b expression (Fig. 3b). Compared with PBS treatment (group C), 8-week DT treatment drastically decreased the number of GFP^+^Collagen-I^+^ and GFP^+^ fibronectin^+^ neoplastic fibrocytes in Jak2V617F/CD11b-DTR mice (group D), but their number was still greater than that in CD11b-DTR mice treated with PBS (group A) (Fig. 3b, c). These fibrocytes in Jak2V617F/CD11b-DTR mice treated with PBS (group C) also expressed CD45.2 and CD68 (Supplementary Fig. 8). The number of Gli1^+^Collagen-I^+^ and LepR^+^Collagen-I^+^ myofibroblasts was also greater in Jak2V617F/CD11b-DTR mice treated with PBS (group C) than in CD11b-DTR mice treated with PBS (group A), and DT treatment had a little effect (Fig. 3c). FACS analysis showed that fibrocytes comprised less than 1% of BMCs in CD11b-DTR mice treated with PBS (group A), and DT treatment did not alter this percentage. In Jak2V617F/CD11b-DTR mice treated with PBS (group C), CD45.2^+^Collagen-I^+^, CD11b^+^Collagen-I^+^, and CD68^+^Collagen-I^+^neoplastic fibrocytes comprised up to about 8% of BMCs; DT treatment drastically decreased this percentage but it was still higher than in CD11b-DTR mice treated with PBS (group A), consistent with the immunostaining assay (Fig. 3d, e). Myofibroblasts comprised about 1–1.5% of BMCs from recipient mice transplanted with Jak2V617F/CD11b-DTR mice treated with PBS (group C), and DT treatment decreased this percentage slightly.

**Fig. 4:**
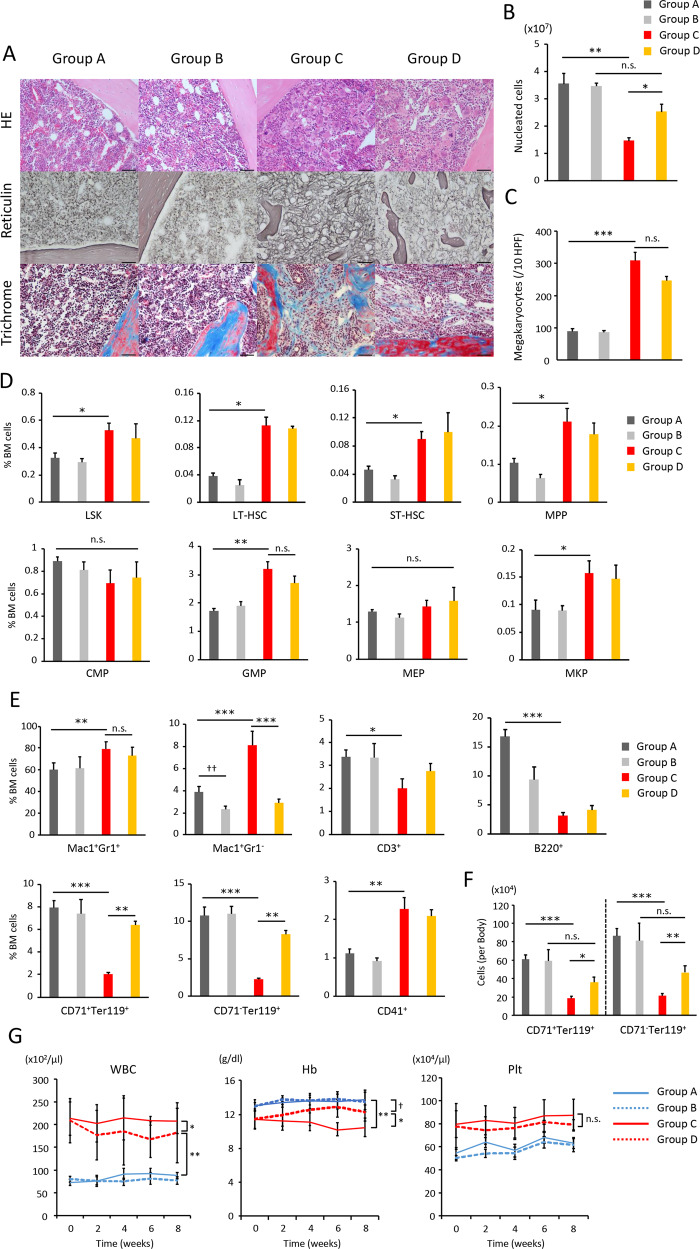
Monocyte depletion ameliorates BM fibrosis and anemia in mice transplanted with Jak2V617F BMCs. **a** Representative images of HE, reticulin silver, and Masson trichrome staining of BM sections from recipient mice transplanted with Jak2V617F/CD11b-DTR BMCs and those transplanted with CD11b-DTR BMCs (*n* = 3 in each group). Bars: (HE) 50 μm; (reticulin) 50 μm; (trichrome) 50 μm. **b** The total numbers of BM nucleated cells in one femur and one tibia. **c** The number of megakaryocytes is presented as the average of 10 random HPFs from three mice of each type. **d** The proportion of LSKs (Lin^−^Sca-1^+^ c-Kit^+^), long-term HSCs (CD150^+^48^−^Lin^−^Sca-1^+^c-Kit^+^), short-term HSCs (CD150^−^48^−^Lin^−^Sca-1^+^c-Kit^+^), MPPs (CD150^−^48^+^Lin^−^Sca-1^+^c-Kit^+^), CMPs (IL-7Rα^−^Lin^−^c-Kit^+^Sca-1^−^FcγRloCD34^+^), GMPs (IL-7Rα^−^Lin^−^c-Kit^+^Sca-1^−^FcγR^+^CD34^+^), MEPs (IL-7Rα^−^Lin^−^c-Kit^+^Sca-1^−^FcγRloCD34^−^), and MKPs (CD9^+^CD41^+^FcγRloc-kit^+^Lin^−^) in BMCs was analyzed by flow cytometry. **e** FACS analysis of Mac1^+^Gr1^+^ granulocytes, Mac1^+^Gr1^−^ monocytes, CD3^+^ T cells, B220^+^ B cells, CD71^+^Ter119^+^ early erythroblasts, CD71^−^Ter119^+^ late erythroblasts, and CD41^+^ megakaryocytes in BM. **f** The absolute number of CD71^+^Ter119^+^ early erythroblasts and, CD71^−^Ter119^+^ late erythroblasts per mouse. Six mice were analyzed in group A and B, and eight mice were analyzed in group C and D (**b**, **d**−**f**). Data are expressed as means ± SEM. One-way ANOVA followed by the Tukey–Kramer test was used (**b**–**f**). ***P < 0.001, **P < 0.01, *P < 0.05, ^☨☨^*P* < 0.01. n.s. not significant. **g** Peripheral blood counts in each group (*n* = 12 in group A and C, *n* = 14 in group B and D) during 8-week treatment with PBS or DT. Data are expressed as means ± SEM. ANOVA with repeated measures was used. **P < 0.01, *P < 0.05, ^☨^*P* < 0.05. n.s. not significant.

**Fig. 5:**
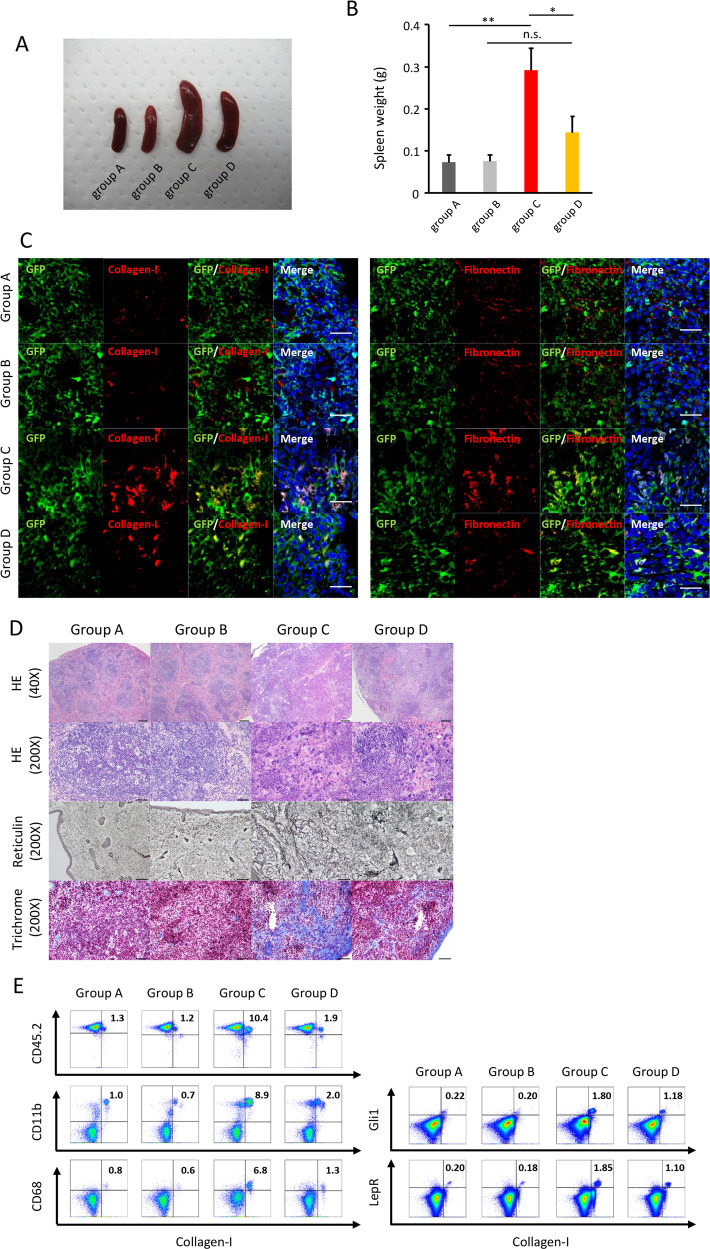
Monocyte depletion ameliorates splenic fibrosis and splenomegaly in mice transplanted with BMCs from Jak2V617F TG. **a** Representative spleens from 8-week PBS-treated recipient mice transplanted with CD11b-DTR BMCs (Group A), 8-week DT-treated recipient mice transplanted with CD11b-DTR BMCs (Group B), 8-week PBS-treated Jak2V617F/CD11b-DTR BMCs (Group C), and 8-week DT-treated Jak2V617F/CD11b-DTR BMCs (Group D). **b** Spleen weight after 8-week PBS or DT treatment (*n* = 6 in group A and B, *n* = 8 in group C and D). Data are expressed as means ± SEM. One-way ANOVA followed by the Tukey–Kramer test was used. **P < 0.01, *P < 0.05. n.s. not significant. **c** Immunofluorescence imaging of spleen sections from each group of recipient mice. The representative result is shown in three independent experiments. GFP was expressed under the CD11b promoter. DAPI staining is shown in blue (merge). Bars: 20 μm. **d** Representative imaging with HE, reticulin silver, and Masson trichrome stains of spleen sections from each group of mice (*n* = 3 in each group). Bars: (HE) 200 µm (top) and 50 µm (second column), (reticulin, trichrome) 50 μm. **e** Representative plots of FACS analysis of CD45.2^+^Collagen-I^+^, CD11b^+^Collagen-I^+^, and CD68^+^Collagen-I^+^ fibrocytes, and Gli1^+^Collagen-I^+^ and LepR^+^Collagen-I^+^ myofibroblasts in spleen cells isolated from each recipient mouse after 8-week PBS or DT treatment. The reproducibility was confirmed by six experiments.

Recipient mice transplanted with Jak2V617F/CD11b-DTR BMCs (group C) developed PMF-like phenotype characterized by fibrosis, decreased cellularity, increased megakaryocytes in BM, anemia, leukocytosis, thrombocytosis in peripheral blood (PB), and massive splenomegaly associated with extramedullary hematopoiesis (Figs. 4 and 5). In BM, the proportion of long- and short-term HSCs, as well as MPPs, GMPs, MKPs, Mac1^+^Gr1^+^ granulocytes, Mac1^+^Gr1^−^ monocytes, and CD41^+^ megakaryocytes increased, while that of CD71^+^Ter119^+^ early erythroblasts and CD71^−^TER119^+^ late erythroblasts decreased (Fig. 4d, e). The absolute number of early and late erythroblasts was calculated by summing the estimated numbers in whole-body BM and spleens [17], and was found to be drastically decreased in Jak2V617F/CD11b-DTR mice (group C) compared with control CD11b-DTR mice (group A) (Fig. 4f). By monocyte depletion, Jak2V617F/CD11b-DTR mice treated with DT (group D) showed not only a decrease in neoplastic fibrocytes in BM, but also the complete elimination of reticulin fibers and a near-complete disappearance of collagen fibers, which together led to a reversal of the decrease in BM cellularity compared with Jak2V617F/CD11b-DTR mice treated with PBS (group C) (Fig. 4a, b). In contrast, neoplastic fibrocyte depletion did not affect the increased megakaryocyte count or the increased proportion of HSCs and progenitors such as MPPs, GMPs, and MKPs (Fig. 4c, d). Consistent with this, neoplastic monocyte depletion only minimally improved leukocytosis and thrombocytosis (Fig. 4g).

Anemia occurred in Jak2V617F/CD11b-DTR mice (group C, D) before starting PBS or DT treatment, and was improved by neoplastic fibrocyte depletion; when these mice were treated with 8-week DT, they still exhibited a little lower hemoglobin values compared with CD11b-DTR mice (group A) (Fig. 4g). The absolute numbers of CD71^+^Ter119^+^ early erythroblasts and CD71^−^Ter119^+^ late erythroblasts in the bodies of these mice were almost normalized by neoplastic monocyte depletion (Fig. 4f).

Splenomegaly is a hallmark of PMF, and Jak2V617F/CD11b-DTR mice (group C) exhibited massive splenomegaly (Fig. 5a, b). An overabundance of neoplastic fibrocytes expressing Collagen-1, fibronectin, CD45.2, GFP (CD11b), and CD68 was observed in the spleens of Jak2V617F/CD11b-DTR mice (group C) (Fig. 5c and Supplementary Fig. 9). Histological examination revealed that the normal splenic architecture was barely recognizable in these mice, and there were significant increases in the numbers of immature cells and reticulin and collagen fibers (Fig. 5d). The proportion of long- and short-term-HSC, as well as MPPs, CMPs, GMPs, MEPs, MKPs, Mac1^+^Gr1^+^ granulocytes, Mac1^+^Gr1^−^ monocytes, CD71^+^Ter119^+^ early erythroblasts, CD71^−^Ter119^+^ late erythroblasts, and CD41^+^ megakaryocytes increased, while those of CD3^+^ T cells decreased (Supplementary Fig. 10). DT treatment drastically, but not completely, reduced the numbers of collagen- and fibronectin-producing cells in spleens (group D) (Fig. 5c, e and Supplementary Fig. 9b, c). Neoplastic monocyte depletion shrank enlarged spleens (Fig. 5a, b). Microscopic analysis revealed an improvement in the damaged spleen architecture and the disappearance of splenic fibrosis (Fig. 5d). The proportion of invading immature hematopoietic cells in spleens was unaffected by neoplastic monocyte depletion, except for the proportion of Mac1^+^Gr1^−^ monocytes and CD71^+^Ter119^+^ early erythroblasts, both of which decreased (Supplementary Fig. 10).

### The effect of TGF-β1 on the differentiation of neoplastic fibrocytes

**Fig. 6:**
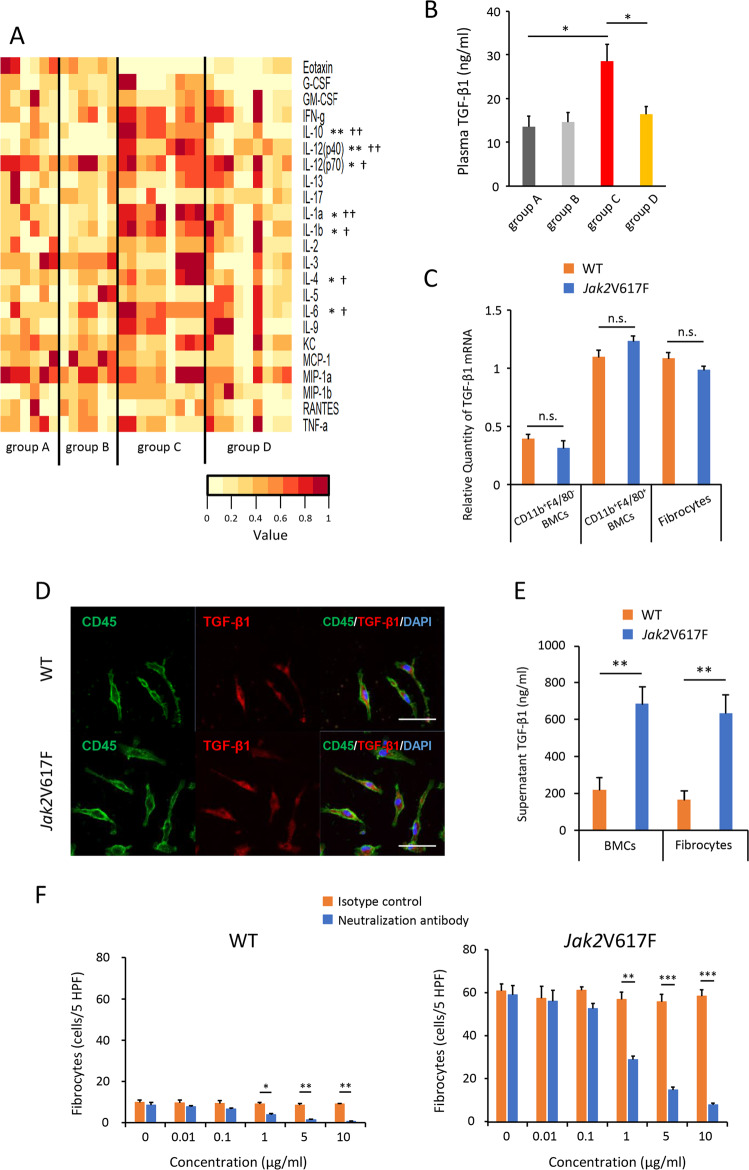
Fibrocytes produce multiple cytokines, including TGF-β1. **a** Heat maps comparing cytokines levels in plasma isolated from recipient mice transplanted with Jak2V617F/CD11b-DTR BMCs and those transplanted with CD11b-DTR BMCs. Six samples in groups A and B, and nine samples in groups C and D were used. A color gradient from white (low levels) to brown (high levels) is shown. One-way ANOVA followed by the Tukey–Kramer test was used. **P < 0.01 and *P < 0.05 (group A vs group C). ^☨☨^*P* < 0.01 and ^☨^*P* < 0.05 (group C vs group D). **b** Plasma TGF-β1 levels are shown for each experimental group (*n* = 6 in group A and B, *n* = 8 in group C and D). Data are expressed as means ± SEM. One-way ANOVA followed by the Tukey–Kramer test was used. *P < 0.05. **c** Quantitative RT-PCR for TGF-β1 in CD11b^+^F4/80^−^ BMCs, CD11b^+^F4/80^+^ BMCs, and cultured fibrocytes from Jak2V617F TG and WT mice. The reproducibility was confirmed by two experiments. **d** Representative immunofluorescence imaging of cultured fibrocytes from BM MNCs of WT mice (top) and Jak2V617F TG (bottom). Bars: 50 μm. **e** Supernatants TGF-β1 levels secreted from 2-day-cultured BMCs and fibrocytes are shown. The reproducibility was confirmed by two experiments. **f** The inhibitory effect of TGF-β1-neutralizing antibodies on neoplastic fibrocyte growth. TGF-β1-neutralizing antibodies or isotype control were added in the culture of BM MNCs from WT (left) and Jak2V617F TG mice (right) mice in conditions that promote differentiation of monocytes to fibrocytes at the beginning of culture. The reproducibility was confirmed by two experiments. Data are expressed as means ± SEM, and the two-tailed student’s *t* test was used (**c**, **e**, **f**). ***P < 0.001, **P < 0.01, *P < 0.05. n.s. not significant.

Circulating cytokine levels are elevated in MF patients, and the distinct clinical features of MF have been attributed in part to dysregulated production of inflammatory cytokines. Plasma cytokine levels were assayed in Jak2V617F/CD11b-DTR mice treated with PBS or DT, in parallel with CD11b-DTR mice as controls (Fig. 6a, b). Of 23 assayed cytokines, the following seven demonstrated significantly higher levels in Jak2V617F/CD11b-DTR mice treated with PBS (group C) than in CD11b-DTR mice treated with PBS (group A): IL-10, IL-12 (P40), IL-12 (P70), IL-1α, IL-1β, IL-4, and IL-6. All these seven cytokines were lowered by monocyte depletion (group D) (Fig. 6a).

The involvement of TGF-β1 in BM fibrosis was previously reported. Plasma TGF-β1 levels were about twofold higher in Jak2V617F/CD11b-DTR mice treated with PBS (group C) than in CD11b-DTR mice treated with PBS (group A), and neoplastic monocyte depletion normalized plasma TGF-β1 levels (group D) (Fig. 6b). TGF-β1 is produced by many kinds of hematopoietic cells, including megakaryocytes, platelets, monocytes, immature myeloid cells, and B cells [18], and, as shown in one study, by fibrocytes [19]. We therefore investigated the production of TGF-β1 from fibrocytes in Jak2V617F-induced PMF. As shown in Fig. 6c, the expression of TGF-β1 mRNA in CD11b^+^F4/80^+^ BMCs composed of monocytes and fibrocytes was greater than that in CD11b^+^F4/80^−^ BMCs composed mainly of granulocytes. Fibrocytes differentiated from BMCs in vitro in 5-day culture also expressed TGF-β1 mRNA. There was no difference in TGF-β1 mRNA expression according to the presence or absence of Jak2V617F mutation. In immunofluorescence staining, most fibrocytes that were differentiated from BMCs in vitro produced TGF-β1 (Fig. 6d). We next examined the secreted TGF-β1 from fibrocytes. Jak2V617F and WT fibrocytes were differentiated from 1 × 10^6^ Jak2V617F and WT BMCs, respectively. The number of differentiated fibrocytes in vitro was about five times greater in dishes with Jak2V617F BMCs than in those with WT BMCs (Supplementary Fig. 11). The supernatant obtained by culturing fibrocytes derived from 1 × 10^6^ BMCs for 2 days contained almost the same amount of TGF-β1 protein as the supernatant obtained by culturing 1 × 10^6^ BMCs for 2 days (Fig. 6e). The amount of TGF-β1 secreted from Jak2V617F fibrocytes was about four times greater than that secreted from WT fibrocytes.

We next examined whether elevated TGF-β1 affected the growth of neoplastic fibrocytes. BM MNCs from WT or Jak2V617F TG mice were cultured in vitro for 5 days with TGF-β1-neutralizing antibodies or control antibodies. The number of cultured neoplastic fibrocytes was decreased by neutralizing antibodies at a concentration of 1 μg/mL; further dose decreases occurred in a dose-dependent manner. This concentration of TGF-β1-neutralizing antibodies also decreased the number of fibrocytes from WT mice, but had little effect on the numbers of CFU-GM and CFU-GEMM obtained from both WT and Jak2V617F BM MNCs (Fig. 6f and Supplementary Fig. 12)

## Discussion

We showed in this study that neoplastic fibrocytes, but not WT myofibroblasts, were primary contributors to the pathogenesis of Jak2V617F-induced MF. BMCs from Jak2V617F TG mice contained many collagen- and fibronectin-producing fibrocytes, and in a competitive transplantation assay using Jak2V617F and WT BMCs, the vast majority of collagen- and fibronectin-producing cells were neoplastic fibrocytes, whereas only a small portion were WT myofibroblasts. Except for the elevated number of megakaryocytes in BM and the leukocytosis and thrombocytosis in PB, all characteristic PMF features, including BM fibrosis, splenomegaly, and anemia, that were observed in recipient mice transplanted with Jak2V617F BMCs, were ameliorated by neoplastic monocyte depletion. Plasma TGF-β1 levels were about twofold higher in recipient mice transplanted with Jak2V617F/CD11b-DTR BMCs than in those transplanted with CD11b-DTR BMCs, and neoplastic monocyte depletion normalized plasma TGF-β1 levels. Neoplastic fibrocyte differentiation from monocytes was increased by TGF-β1.

Fibrocytes are spindle-shaped, fibroblast-like cells that differentiate from monocytes and express markers of both hematopoietic cells (CD34, 43, 45, 68) and extracellular matrix proteins (Collagen-I, Collagen-III, fibronectin, αSMA, vimentin) [19–22]. Fibrocytes play a central role in the pathogenesis of fibrosis in organs such as the lung, kidney, and heart [23–25]. In contrast, since previous studies showed that cultured BM MSCs were polyclonal and did not originate from neoplastic clones, BM fibrosis has been thought to be a reactive event in which TGF-β1 is overproduced by increased numbers of neoplastic megakaryocytes and platelets, causing MSCs to induce fibrosis [3, 4]. Recent studies suggested that fibrocytes originated from hematopoietic cells in BM fibrosis [10, 11].

Fibrocytes differentiate from monocytes and make up <1% of BMCs in WT mice [19]. In Jak2V617F TG mice, the number of fibrocytes was increased, and these cells comprised about 8% of BMCs. These results are consistent with a previous report by Verstovsek et al. showing that BM from PMF patients contained numerous fibrocytes [10]. To confirm that the increased fibrocytes in this study were neoplastic clones, we transplanted Jak2V617F BMCs together with competitor WT BMCs into irradiated WT recipient mice. The recipient mice developed PMF-like phenotype, and their BM contained many CD45.2^+^Collagen-I^+^ and CD45.2^+^fibronectin^+^ cells, indicating that neoplastic fibrocytes derived from Jak2V617F cells were increased in number in Jak2V617F-induced MF. WT myofibroblasts were also increased in number, although to a lesser degree. These results showed that neoplastic fibrocytes were the primary contributors to BM fibrosis induced by Jak2V617F, with WT myofibroblasts playing a lesser role.

Manshouri et al. transplanted CD14^+^CD34^−^ monocytes from PMF patients into immunodeficient mice [26]; these mice subsequently developed PMF-like features, indicating that neoplastic monocytes are sufficient for the development of a short period of BM fibrosis and splenomegaly. Consistent with these observations, the present study showed that neoplastic monocyte depletion by 8-week DT administration to recipient mice transplanted with Jak2V617F/CD11b-DTR BMCs induced the resolution of BM fibrosis, along with a drastic decrement of the number of neoplastic fibrocytes, and, to a lesser extent, the number of WT myofibroblasts. These results also confirmed the above finding that neoplastic fibrocytes harboring Jak2V617F produced collagen and fibronectin in Jak2V617F-induced MF. This is consistent with a previous study that demonstrated the usefulness of therapy targeting fibrocytes [10]. In that study, xenograft mice transplanted with BM MNCs from PMF patients developed BM fibrosis, and treatment with the fibrocyte inhibitor serum amyloid P prolonged survival and slowed fibrosis development.

Neoplastic fibrocyte depletion ameliorated not only BM fibrosis but also the anemia and splenomegaly induced by Jak2V617F. The increases in BM cellularity and the absolute numbers of erythroid progenitors induced by neoplastic fibrocyte depletion might have contributed to the improvement of anemia. Neoplastic monocyte depletion in recipient mice transplanted with Jak2V617F/CD11b-DTR BMCs reduced the elevated levels of inflammatory cytokines such as IL-12, IL-1α, and IL-1β, which might also have helped resolve anemia [27]. Extramedullary hematopoiesis is generally acknowledged to be the cause of splenomegaly associated with PMF, but neoangiogenesis was also shown to contribute [28]. In recipient mice transplanted with Jak2V617F/CD11b-DTR BMCs, monocyte depletion resulted in about a two-third reduction in spleen weight as well as pathological improvement of the damaged splenic architecture. This depletion also induced not only the eradication of fibrocytes in the enlarged spleen, but also a reduction of elevated inflammatory cytokines such as IL-1α, IL-1β, and IL-6. These cytokines have been reported to promote neoangiogenesis [29, 30] and also to induce splenomegaly in vivo [31–33]. The reduction of inflammatory cytokines by monocyte depletion might contribute to the improvement of splenomegaly by ameliorating the expanded capillary vascularization in recipient mice transplanted with Jak2V617F/CD11b-DTR BMCs.

In contrast to the extensive improvement of BM fibrosis and anemia achieved by neoplastic monocyte depletion, megakaryocytic and granulocytic lineage cells were only minimally affected. These observations are reasonable since both lineages of cells differentiate from HSCs [34]. These findings also suggest that the increase in the number of neoplastic megakaryocytes did not play a major role in the pathogenesis of BM fibrosis.

Even though we demonstrated the essential role of neoplastic fibrocytes in BM fibrosis induced by Jak2V617F, our findings could not rule out the involvement of myofibroblasts in BM fibrosis. BMCs from Jak2V617F TG also consisted partially of collagen- and fibronectin-producing myofibroblasts, in numbers that were 7–10 times greater than those in WT BMCs. In recipient mice transplanted with Jak2V617F/CD11b-DTR BMCs, these collagen- and fibronectin-producing myofibroblasts were decreased by monocyte depletion, but only to a small degree, and the proportion of fibrocytes after monocyte depletion was still much higher than that in recipient mice transplanted with CD11b-DTR BMCs. These collagen- and fibronectin-producing myofibroblasts might contribute slightly to BM fibrosis.

The involvement of TGF-β1 in BM fibrosis was previously reported [8, 9]. For example, recipient mice transplanted with TGF-β1-deficient BMCs that were retrovirally transfected with thrombopoietin (TPO) did not develop MF, in contrast to recipient mice transplanted with WT BMCs retrovirally transfected with TPO [35]. Plasma TGF-β1 levels were about twofold higher in recipient mice transplanted with Jak2V617F/CD11b-DTR BMCs, which developed PMF, than in recipient mice transplanted with CD11b-DTR BMCs, which did not develop PMF. Neoplastic monocyte depletion normalized plasma TGF-β1 levels, and also drastically reduced the number of neoplastic fibrocytes but did not affect the numbers of megakaryocytes or platelets, which were previously thought to be the main source of TGF-β1 in PMF [36]. These observations indicate that neoplastic monocytes and the fibrocytes that differentiate from them are likely to be responsible for the elevated plasma TGF-β1 level in Jak2V617F-induced MF. Fibrocytes differentiated from Jak2V617F BMCs produced TGF-β1 in this study, in agreement with a previous report [37], and might contribute to the higher plasma TGF-β1 level in Jak2V617F-induced MF. It is currently uncertain whether monocytes or fibrocytes are the major source of TGF-β1 in Jak2V617F-induced MF. We could not directly compare the amount of either TGF-β1 mRNA or secreted protein between monocytes and fibrocytes, as BMCs were freshly prepared from mice whereas fibrocytes were differentiated from BMCs in vitro in 5-day culture in our experiment, and CD11b^+^F4/80^+^ BMCs were composed of monocytes and fibrocytes. Even though the expression of TGF-β1 mRNA was comparable despite their different cell origins (WT and Jak2V617F clones, respectively), the amount of secreted TGF-β1 protein in supernatants from cultured Jak2V617F BMCs and fibrocytes was greater than that in supernatants from cultured WT BMCs and fibrocytes, respectively. These differences in TGF-β1 production might be due to the discrepancy in the proportion of monocytes and fibrocytes in BMCs between WT mice and Jak2V617F TG mice, and might account for the twofold difference in plasma TGF-β1 levels between them. It should be noted that our study was inconsistent with a previous report that found no difference in the level of TGF-β1 secreted by fibrocytes of PMF patients versus controls [10]. An extensive investigation of the source of elevated TGF-β1 in PMF is required, using both patient samples and mouse models.

**Fig. 7:**
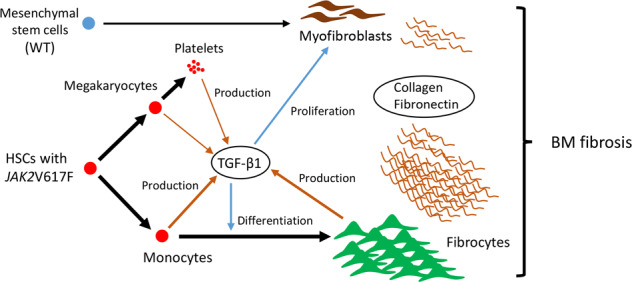
The mechanism of fibrosis in PMF (schematic diagram). TGF-β1 is produced by not only megakaryocytes and platelets but also monocytes and fibrocytes. While TGF-β1 promotes the proliferation of wild-type myofibroblast, it strongly promotes the differentiation of neoplastic monocytes having JAK2V617F mutation into fibrocytes. The proportion of neoplastic fibrocyte in collagen- and fibronectin-producing cells is much higher than that of wild-type myofibroblast.

TGF-β1 was reported to play a role in fibrocyte differentiation [38], and in our case TGF-β1 supported the differentiation of neoplastic fibrocytes from neoplastic monocytes, since neutralizing antibodies against TGF-β1 decreased the number of fibrocytes from Jak2V617F BM MNCs in vitro. The elevated TGF-β1 level observed in Jak2V617F/CD11b-DTR mice might have caused neoplastic monocytes to differentiate into fibrocytes. If so, the effect of TGF-β1 is likely to be restricted to neoplastic clones, because no increment of WT fibrocytes was observed. TGF-β1 has previously been thought to stimulate nearby myofibroblasts to proliferate and produce collagen. Since collagen- and fibronectin-producing myofibroblasts were present in Jak2V617F/CD11b-DTR mice to a lesser extent, higher TGF-β1 levels might also have affected myofibroblast proliferation, and the number of myofibroblasts decreased with decreasing levels of TGF-β1 (Fig. 7).

Our findings indicated that neoplastic fibrocytes played an essential role in BM fibrosis induced by Jak2V617F, and mesenchymal myofibroblasts contributed more minimally. TGF-β1 was required for the greater fibrocyte differentiation from neoplastic monocytes. Ruxolitinib treatment, currently the standard therapy for advanced MF patients, drastically decreases splenomegaly and resolves MF-associated symptoms, but the drug rarely resolves BM fibrosis or anemia [5, 6, 39]. Since neoplastic monocyte depletion successfully improved BM fibrosis and anemia in this study, supplementation of ruxolitinib with therapy targeting neoplastic monocytes is a promising approach.

## Supplementary information

Revised Supplementary Information_final
