# Supplementary material for: Neoplastic fibrocytes play an essential role in bone marrow fibrosis in Jak2V617F-induced primary myelofibrosis mice
Source: Leukemia. 2020 May 29;35(2):454–67. doi: 10.1038/s41375-020-0880-3 (PMC7862060; doi:10.1038/s41375-020-0880-3)
Supplement: Supplementary file 1 — Revised Supplementary Information_final [file 41375_2020_880_MOESM1_ESM.docx]

**Supplementary Methods**

**Animals and disease models**

B6 mice congenic for the CD45.1 locus (B6-CD45.1) were purchased from Sankyo Laboratory Service (Tokyo, Japan). We adopted three kinds of recipient mice as disease models: 9 Gy-irradiated 8-week-old wild-type (WT) recipient mice transplanted with 1×10^6^ fetal liver cells (FLCs) from *Jak2*V617F transgenic (TG) or WT mice; WT recipient mice (CD45.1) transplanted with 5×10^6^ bone marrow (BM) cells (BMCs) from *Jak2*V617F TG (CD45.2) or WT mice (CD45.2), together with 1×10^6^ BMCs from WT mice (CD45.1); and WT recipient mice (CD45.1) transplanted with BMCs from CD11b-DTR TG (CD45.2) or *Jak2*V617F/CD11b-DTR TG (CD45.2). To deplete monocytes *in vivo*, at 8 weeks after transplantation, recipient mice transplanted with BMCs from CD11b-DTR TG or *Jak2*V617F/CD11b-DTR TG were randomized into two groups and administered 100 μl phosphate buffered saline (PBS) or 15 ng/g diphtheria toxin (DT) (List Biological Labs, Campbell, CA, USA) diluted with 100 μl PBS by intraperitoneal injection every 2 days for 8 weeks.

***In vitro* fibrocyte differentiation assay**

BMCs were collected from *Jak2*V617F TG and WT mice by flushing two femurs and two tibias and diluted with PBS. Then, mononuclear cells (MNCs) were isolated by Lympholyte-Mammal (Cedarlane, Burlington, NC, USA) at 800 × g for 20 minutes at room temperature. MNCs were collected from the interface, mixed with 10 ml of PBS, and collected by centrifugation at 300 × g for 10 minutes. MNCs were resuspended in PBS and collected by centrifugation, and then resuspended in FibroLife (Lifeline Cell Technology, Oceanside, CA, USA) supplemented with 20 mM 4-(2-hydroxyethyl)-1- piperazineethanesulfonic acid (HEPES) (Nacalai Tesque, Kyoto, Japan), 2 × Non-essential Amino Acids (NEAA) (Wako Pure Chemical Industries, Osaka, Japan), 2 mM sodium pyruvate (Wako Pure Chemical Industries), 4 mM glutamine (Thermo Fisher Scientific, Carlsbad, CA, USA), 100 U/ml penicillin (Wako Pure Chemical Industries), 100 μg/ml streptomycin (Wako Pure Chemical Industries), and 2 × ITS-3 (Sigma-Aldrich, St. Louis, MO, USA). MNCs were cultured in flat-bottomed 24-well tissue culture plates and on micro cover glasses (φ22 mm, Matsunami Glass Industries, Osaka, Japan) in 500-μl volumes at 2.5 × 10^5^ cells per ml in a humidified incubator containing 5% CO_2_ at 37°C.

After 5 days, images of cells in 24-well plates were captured. Then, the cells on cover glasses were fixed in 4% paraformaldehyde (Wako Pure Chemical Industries). After several rinses in PBS, these cells were incubated in 5% normal goat serum/1% bovine serum albumin in PBS for 20 minutes, then incubated with the primary antibodies specific for CD45 (Abcam, Cambridge, MA, USA, ab25386), CD11b (Abcam, ab8878), CD34 (Abcam, ab8158), CD16 (Abcam, ab25235), CD68 (Abcam, ab125212), Collagen-I (Abcam, ab6308, ab34710), Fibronectin (Abcam, ab2413), Alpha SMA (Abcam, ab5694), Vimentin (Abcam, ab45939), CD90 (Abcam, ab3105), Gli1 (Abcam, ab49314), LepR (Invitrogen, Carlsbad, CA, USA, PA5-85621), TGF-β1 (Abcam, ab92486) listed in Supplementary Table 1 for 1-3 hours at room temperature. Slides were then washed in PBS and incubated with the fluorescent secondary antibodies: anti-rat Alexa Fluor 488 (Abcam, ab150157), anti-rabbit Alexa Fluor 488 (Abcam, ab150077), anti-mouse Alexa Fluor 555 (Abcam, ab150114), anti-rabbit Alexa Fluor 555 (Abcam, ab150078) for 30 minutes at room temperature. These sections were then rinsed in PBS and mounted on coverslips with the use of Vectashield with 4’,6-diamidino-2-phenylindole (DAPI) (Vector Laboratories, Burlingame, CA, USA). Microscopy images were acquired using a Zeiss LSM 700 confocal microscope (Athens, GA, USA).

**MSC culture assay**

BM MNCs from *Jak2*V617F TG and WT mice (1 x 10^6^ cells/mL) were cultured in Dulbecco’s Modified Eagle’s Medium (Invitrogen) supplemented with 20% FBS (Sigma-Aldrich**)**, 4 mM glutamine (Thermo Fisher Scientific), 100 U/ml penicillin (Wako Pure Chemical Industries), and 100 μg/ml streptomycin (Wako Pure Chemical Industries), that supports mesenchymal stromal cells (MSCs) proliferation.

**Pathological and** **immunohistochemical examination**

For histological evaluation, tissue samples were fixed in formalin, paraffin embedded, and cut for hematoxylin-eosin staining, reticulin silver staining, and Masson trichrome staining according to standard protocols. For immunofluorescence studies, deparaffinized BM and spleen sections were rehydrated, then boiled in citrate buffer (Abcam) for 10 minutes to retrieve the antigenic sites. After several rinses in PBS, the sections were incubated in 5% normal goat serum/1% bovine serum albumin in PBS for 20 minutes. Slides were then incubated with the primary antibodies listed in Supplemental Table 1 at a predetermined concentration for 12-24 hours at 4℃ in the case of monoclonal antibodies specific for CD45 (Abcam, ab25386), CD45.2 (BioLegend, San Diego, CA, USA, #109802), CD45.1 (Abcam, ab25078), CD11b (Abcam, ab8878), CD68 (Abcam, ab53444), Collagen-I (Abcam, ab6308), Fibronectin (Abcam, ab6328), or for 1 hour at room temperature in the case of polyclonal antibodies specific for Collagen-I (Abcam, ab34710), Fibronectin (Abcam, ab2413), Gli1 (Abcam, ab49314), LepR (Invitrogen, PA5-85621). Slides were then washed in PBS and incubated with fluorescent secondary antibodies: anti-rat Alexa Fluor 488 (Abcam, ab150157), anti-rabbit Alexa Fluor 555 (Abcam, ab150078), anti-rat Alexa Fluor 405 (Abcam, ab175671) for 1 hour at room temperature. Mouse monoclonal antibodies (CD45.2 and Collagen-I) were conjugated to DyLight 405 and 488 using the DyLight system (Abcam, ab201798, ab201799). Mouse monoclonal antibodies (CD45.1 and Fibronectin) were conjugated to DyLight 488 using the DyLight system (Abcam, ab201799). These sections were then rinsed in PBS and mounted on coverslips with the use of Vectashield with DAPI (Vector Laboratories, H-1200) or Vectashield alone (Vector Laboratories, H-1000). Microscopy images were acquired using a Zeiss LSM 700 confocal microscope (Athens). The investigators were blinded to the group allocation during histological examination.

**Fluorescence-activated cell sorter (FACS) analysis**

PB cells were hemolyzed and washed in PBS containing 1% FBS, and blocked with Fc-block (BioLegend, San Diego, CA) for 4 minutes on ice. BMCs and spleen cells suspensions were prepared as previously described^1^. Aliquots of 1 x 10^6^ cells were stained with the conjugated monoclonal antibodies listed in Supplementary Table 2 in PBS plus 1% FBS for 40 minutes on ice. After washing, cells were resuspended in PBS plus 1% FBS and analyzed using a FACS Canto II cytometer (BD Biosciences, San Jose, CA, USA). LSK (Lin^−^Sca-1^+^c-Kit^+^) cells, long-term HSCs (CD150^+^48^−^ Lin^−^Sca-1^+^c-Kit^+^), short-term HSCs (CD150^−^48^−^Lin^−^Sca-1^+^c-Kit^+^), MPPs (CD150^−^48^+^ Lin^−^Sca-1^+^c-Kit^+^), CMPs (IL-7Rα^−^Lin^−^c-Kit^+^Sca-1^−^FcγR^lo^CD34^+^), GMPs (IL-7Rα^−^Lin^−^c-Kit^+^Sca-1^−^FcγR^+^CD34^+^), MEPs (IL-7Rα^−^Lin^−^c-Kit^+^Sca-1^−^FcγR^lo^CD34^−^), and MKPs (CD9^+^CD41^+^FcγR^lo^c-kit^+^Lin^−^) in the BM and spleen were analyzed. Data were analyzed using FlowJo software (Tree Star, Ashland, OR). Cell sorting of CD11b^+^F4/80^+^ BMCs and CD11b^+^F4/80^−^ BMCs was performed using FACSAria II (BD Biosciences).

For intracellular staining, cells were permeabilized with a BD Cytofix/Cytoperm Kit (BD Biosciences, San Diego, CA, USA) and stained with anti-collagen I antibody (R1038B; Rockland, Gilbertsville, Pennsylvania, USA). Viability was tracked using the Fixable Viability Dye eFluor 660 (Invitrogen).

**Quantitative reverse transcriptase PCR**

Total RNA was extracted from sorted cells using an RNeasy Mini Kit (QIAGEN, Hilden, Germany) or by adding Buffer RLT (QIAGEN) directly to the fibrocytes which were differentiated from BMCs *in vitro*.

Complementary DNA was generated using a QuantiTect Reverse Transcription Kit (QIAGEN). Quantitative PCR was performed on a LightCycler 480 (Roche, Welwyn Garden City, UK) and analyzed with the associated software. We used the Roche UPL probes system (Roche) in combination with gene-specific primer sets. The primer sequences and probes were as follows: *Tgfb1*, 5’-tggagcaacatgtggaactc-3’, 5’-gtcagcagccggttacca-3’, and universal probe #72. Gene expression levels were normalized to *Gapdh* using Universal ProbeLibrary Mouse GAPD Gene Assay (Roche).

**Cytokine measurement**

Levels of cytokines were detected using a Bio‑Plex Pro Mouse Cytokine Group I Panel 23‑Plex (#M60009RDPD; BioRad, Hercules, CA, USA), which included 23 cytokines (IL-1α, IL-1β, IL-2, IL-3, IL-4, IL-5, IL-6, IL-9, IL-10, IL-12 (p40), IL-12 (p70), IL-13, IL-17A, eotaxin, G-CSF, IFN-γ, KC, MCP-1, MIP-1α, MIP-1β, RANTES, and TNF-α). The levels of these cytokines were calculated using the flow cytometry-based Luminex 200 system (Luminex Corporation, Austin, TX, USA) and XPONENT software for LX100/LX200 (version 3.1).

**Measurement of secreted TGF-β1 in culture medium**

BMCs (1×10^6^ cells/well) from *Jak2*V617F TG and WT mice were cultured in flat-bottomed 24-well tissue culture plates in RPMI (Invitrogen) supplemented with 2×NEAA (Wako Pure Chemical Industries), 2 mM sodium pyruvate (Wako Pure Chemical Industries), 100 U/ml penicillin (Wako Pure Chemical Industries), and 100 μg/ml streptomycin (Wako Pure Chemical Industries). After 2 days, the supernatants were collected. Fibrocytes were differentiated from BMCs (1×10^6^ cells/well) in the conditions described above in the section titled “*In vitro* fibrocyte differentiation assay.” After 5-day culture, the number of adherent cells with an elongated spindle-shaped morphology was considered to represent the number of fibrocytes. The cells were washed twice with PBS, and then fresh media was added. Two days after the medium change, the supernatants were collected. Measurement of TGF-β1 secreted by BMCs and fibrocytes into the supernatants was performed with murine Quantikine Kits (R&D Systems, Minneapolis, MN, USA) according to the manufacturer’s protocol.

**The evaluation of TGF-β1 on cell growth**

To evaluate the effects of TGF-β1 on fibrocyte differentiation and growth, the indicated concentrations of neutralizing antibodies directed against TGF-β1 (R&D Systems, MAB1835) or isotype antibodies (IgG1) (R&D Systems, MAB002) were added to tissue culture plates at the beginning of culture. After 5 days, the number of CD45^+^Collagen-I^+^ cells was counted.

To detect granulocyte-macrophage colony-forming units (CFUs) (CFU-GM) and granulocyte, erythrocyte, monocyte and megakaryocyte CFUs (CFU-GEMM), 2×10^4^ BM MNCs were plated in methylcellulose (M3434; StemCell Technologies Inc, Vancouver, BC, Canada) containing 3 U/ml erythropoietin, 10 ng/ml recombinant murine IL-3, 10 ng/ml recombinant murine IL-6, and 50 ng/ml recombinant murine stem cell factor according to the manufacturer’s protocols. To evaluate the toxicity to hematopoietic cells, the indicated concentrations of neutralizing antibodies against TGF-β1 (R&D Systems) were added in methylcellulose from the start of the culture. After 7 days, the number of colonies were counted.

**Statistical analysis**

Results are presented as means ± SEM. To assess the statistical significance between pairs of groups, the two-tailed Student’s t-test was used. For multiple group comparison, analysis of variance with *post hoc* correction was used. For the comparison of the hematological values between groups, analysis of variance with repeated measures was used.

**Reference**

1. Shide K, Kameda T, Yamaji T, Sekine M, Inada N, Kamiunten A, et al. Calreticulin mutant mice develop essential thrombocythemia that is ameliorated by the JAK inhibitor ruxolitinib. Leukemia. 2017;31(5):1136-1144.

**Supplementary Table 1. Antibodies used in immunofluorescence analysis**

| Name | Class | Company | Catalog number |
| --- | --- | --- | --- |
| CD45 | Monoclonal | Abcam | ab25386 |
| CD45.2 | Monoclonal | BioLegend | #109802 |
| CD45.1 | Monoclonal | Abcam | ab25078 |
| CD11b | Monoclonal | Abcam | ab8878 |
| CD34 | Monoclonal | Abcam | ab8158 |
| CD16 | Monoclonal | Abcam | ab25235 |
| CD68 | Monoclonal | Abcam | ab53444 |
| CD68 | Polyclonal | Abcam | ab125212 |
| Collagen-Ⅰ | Monoclonal | Abcam | ab6308 |
| Collagen-Ⅰ | Polyclonal | Abcam | ab34710 |
| Fibronectin | Monoclonal | Abcam | ab6328 |
| Fibronectin | Polyclonal | Abcam | ab2413 |
| Alpha SMA | Polyclonal | Abcam | ab5694 |
| Vimentin | Polyclonal | Abcam | ab45939 |
| CD90 | Monoclonal | Abcam | ab3105 |
| Gli1 | Polyclonal | Abcam | ab49314 |
| LepR | Polyclonal | Invitrogen | PA5-85621 |
| TGF-β1 | Polyclonal | Abcam | ab92486 |
| Alexa Fluor 488 rat IgG | Polyclonal | Abcam | ab150157 |
| Alexa Fluor 488 rabbit IgG | Polyclonal | Abcam | ab150077 |
| Alexa Fluor 555 mouse IgG | Polyclonal | Abcam | ab150114 |
| Alexa Fluor 555 rabbit IgG | Polyclonal | Abcam | ab150078 |
| Alexa Fluor 405 rat IgG | Polyclonal | Abcam | ab175671 |

**Supplementary Table 2. Antibodies used in FACS analysis**

| Fluorescence | Antibody | Company | Catalog number |
| --- | --- | --- | --- |
| FITC | F4/80 | BioLegend | #123108 |
| FITC | CD3 | BioLegend | #100204 |
| FITC | CD68 | BioLegend | #137006 |
| FITC | CD34 | eBioscience | #4276888 |
| FITC | CD41 | BioLegend | #133904 |
| FITC | Mac1 | BioLegend | #101206 |
| FITC | B220 | BioLegend | #103206 |
| FITC | CD71 | BioLegend | #113806 |
| FITC | Leptin Receptor | Bioss | bs-0961R |
| FITC | Gli1 | NOVUS | NB600-600AF488 |
| FITC | Rabbit IgG isotype control | abcam | ab37406 |
| PE | CD11b | BioLegend | #101208 |
| PE | Gr-1 | BioLegend | #108408 |
| PE | CD3 | BioLegend | #100206 |
| PE | Ter119 | BioLegend | #116208 |
| PE | Sca-1 | BioLegend | #108108 |
| PE | CD9 | BioLegend | #124806 |
| APC | B220 | BioLegend | #103212 |
| APC | c-Kit | BioLegend | #105812 |
| PerCP-Cy5.5 | Gr-1 | BioLegend | #108428 |
| PerCP-Cy5.5 | streptavidin | BioLegend | #405214 |
| PE-Cy7 | CD16/32 | BioLegend | #101318 |
| PE-Cy7 | Ter119 | BioLegend | #116222 |
| APC-Cy7 | CD45.2 | BioLegend | #109824 |
| APC-Cy7 | CD45 | BioLegend | #103116 |
| Biotin | Collagen-Ⅰ | Rockland | R1038B |
| Biotin | CD3 | BioLegend | #100304 |
| Biotin | CD4 | BioLegend | #100404 |
| Biotin | CD8 | BioLegend | #100704 |
| Biotin | B220 | BioLegend | #103204 |
| Biotin | Mac-1 | BioLegend | #101204 |
| Biotin | Gr-1 | BioLegend | #108404 |
| Biotin | Ter119 | BioLegend | #116204 |
| Biotin | Rabbit IgG isotype control | Abcam | ab200208 |

**
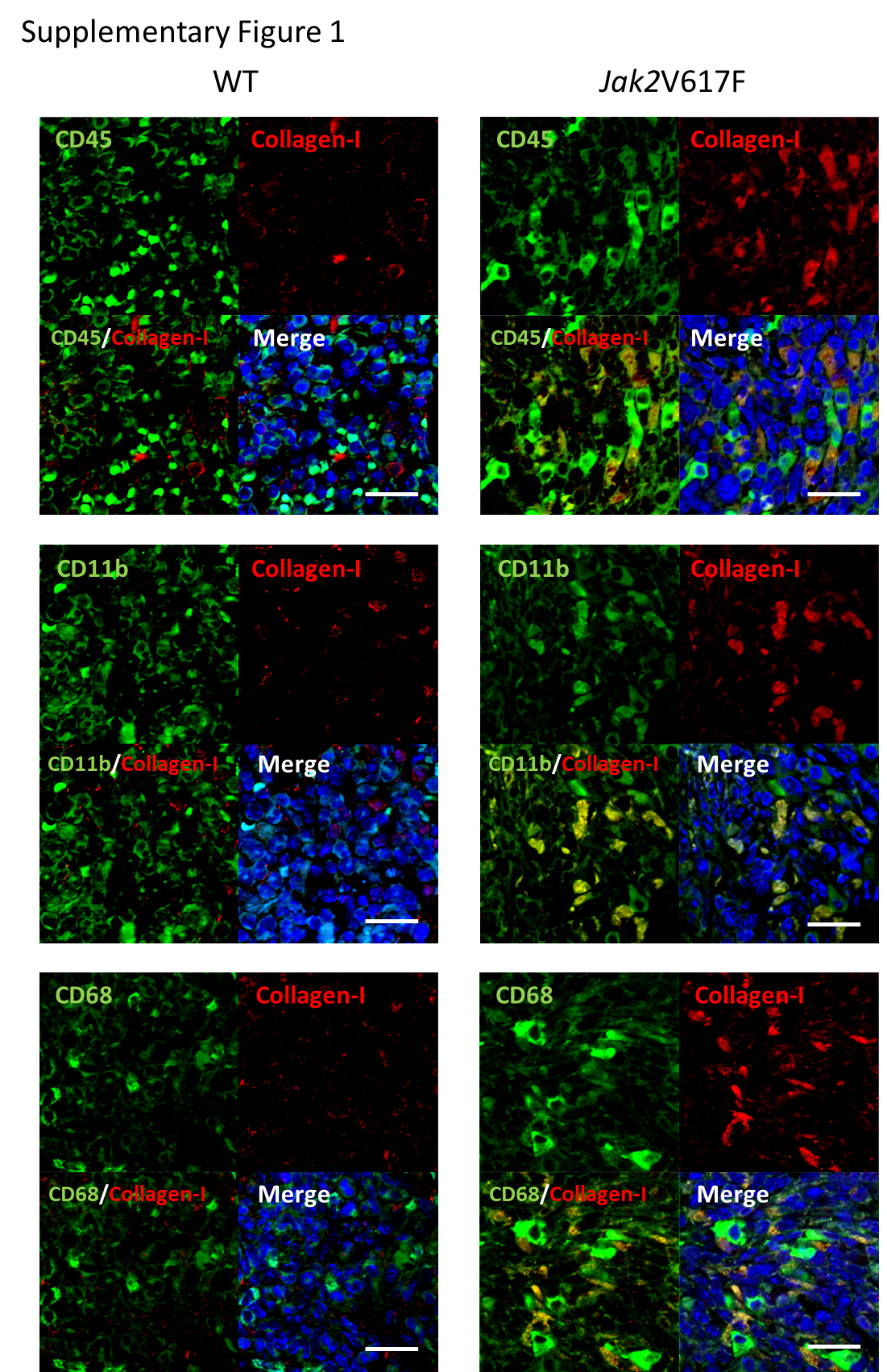
**

**
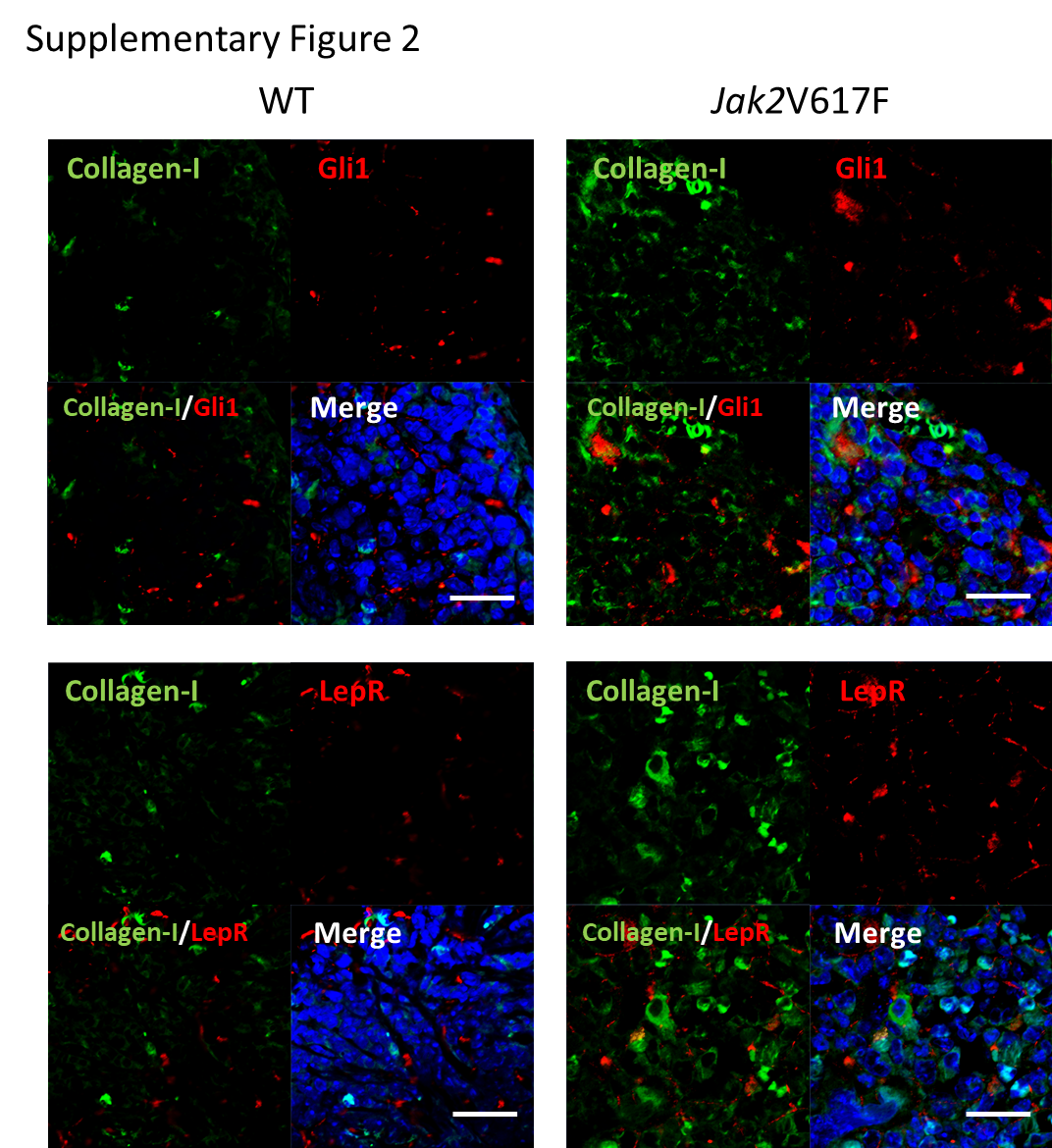
**

**
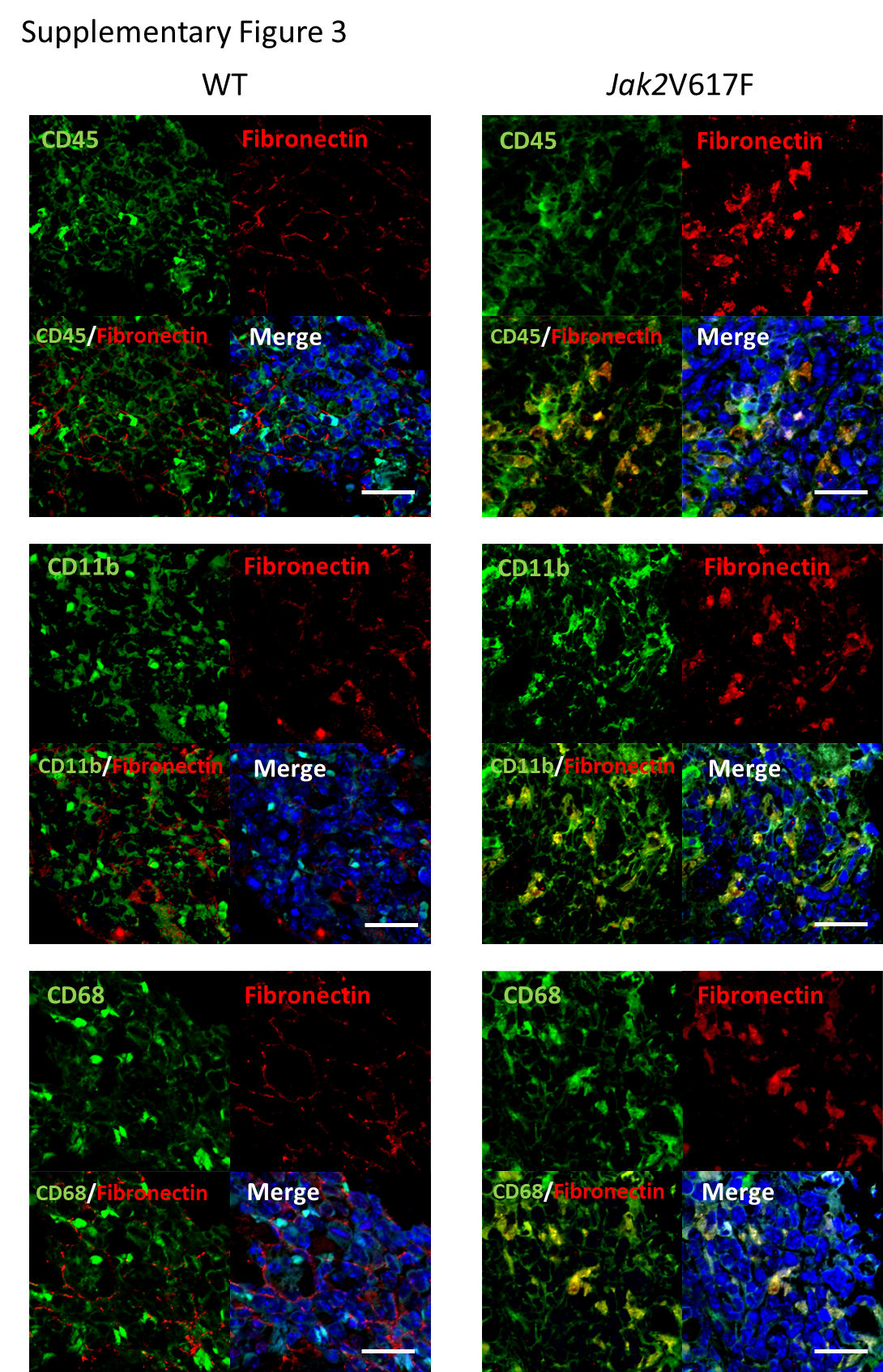
**

**
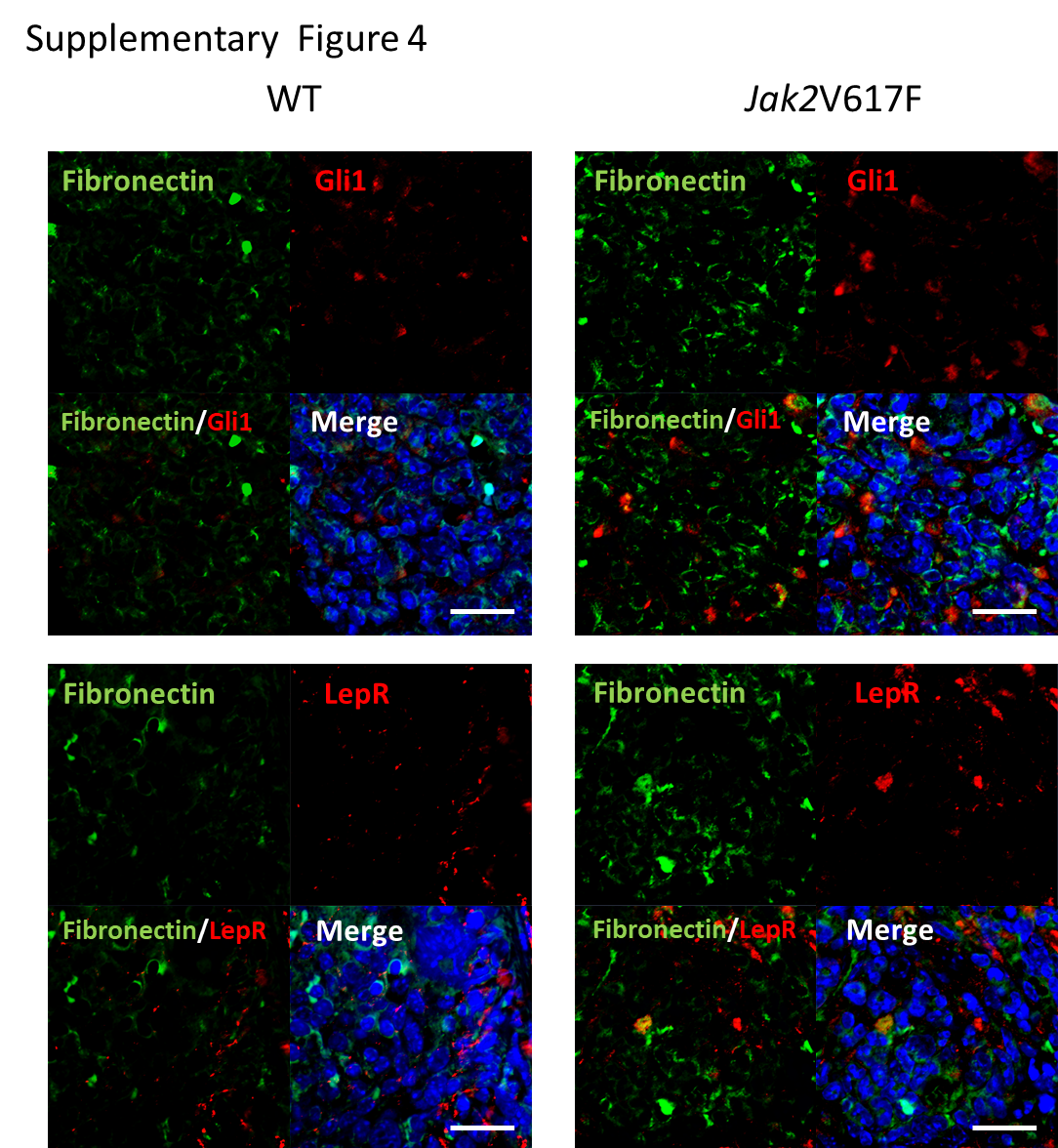
**

**
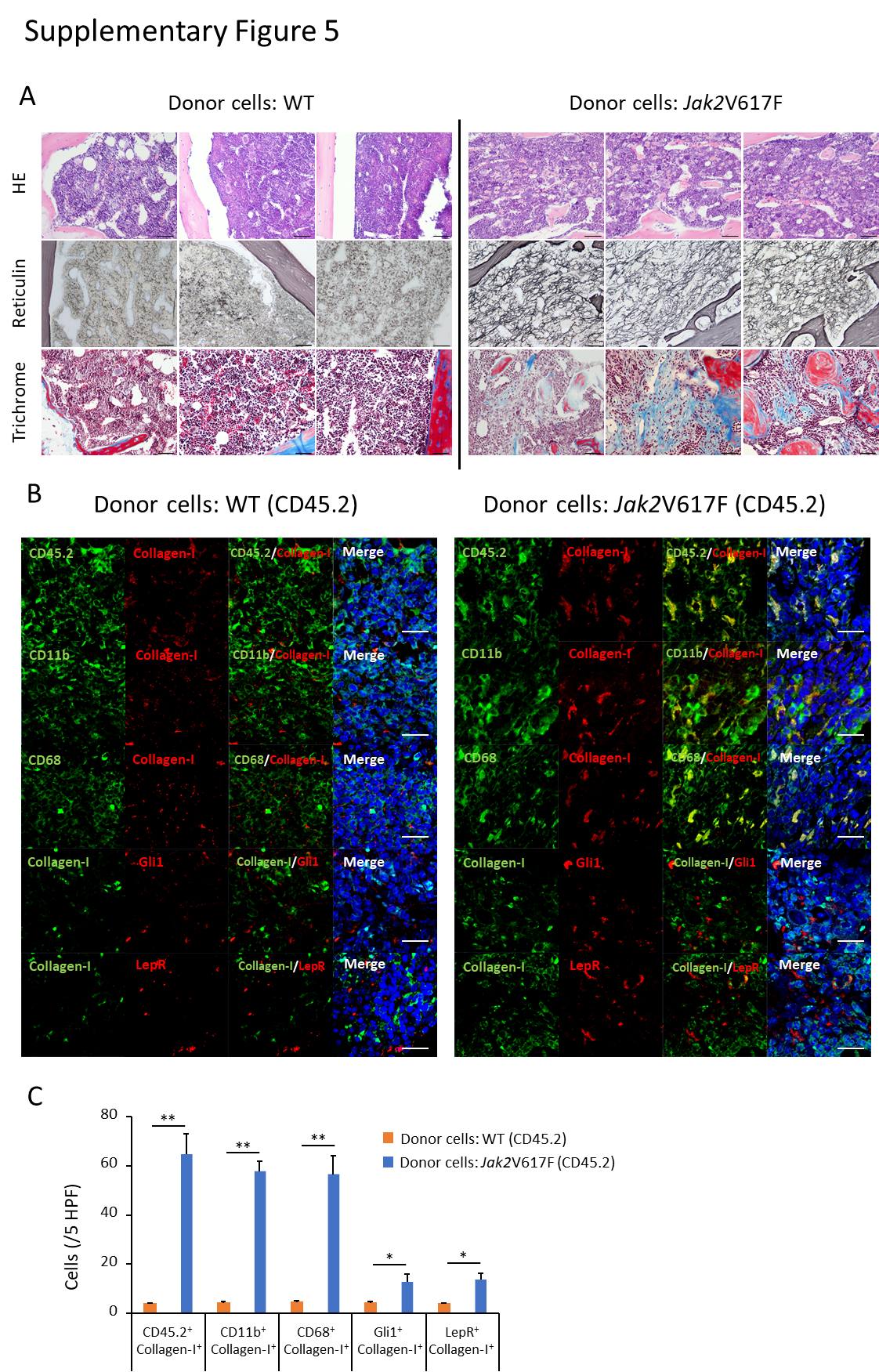
**

**
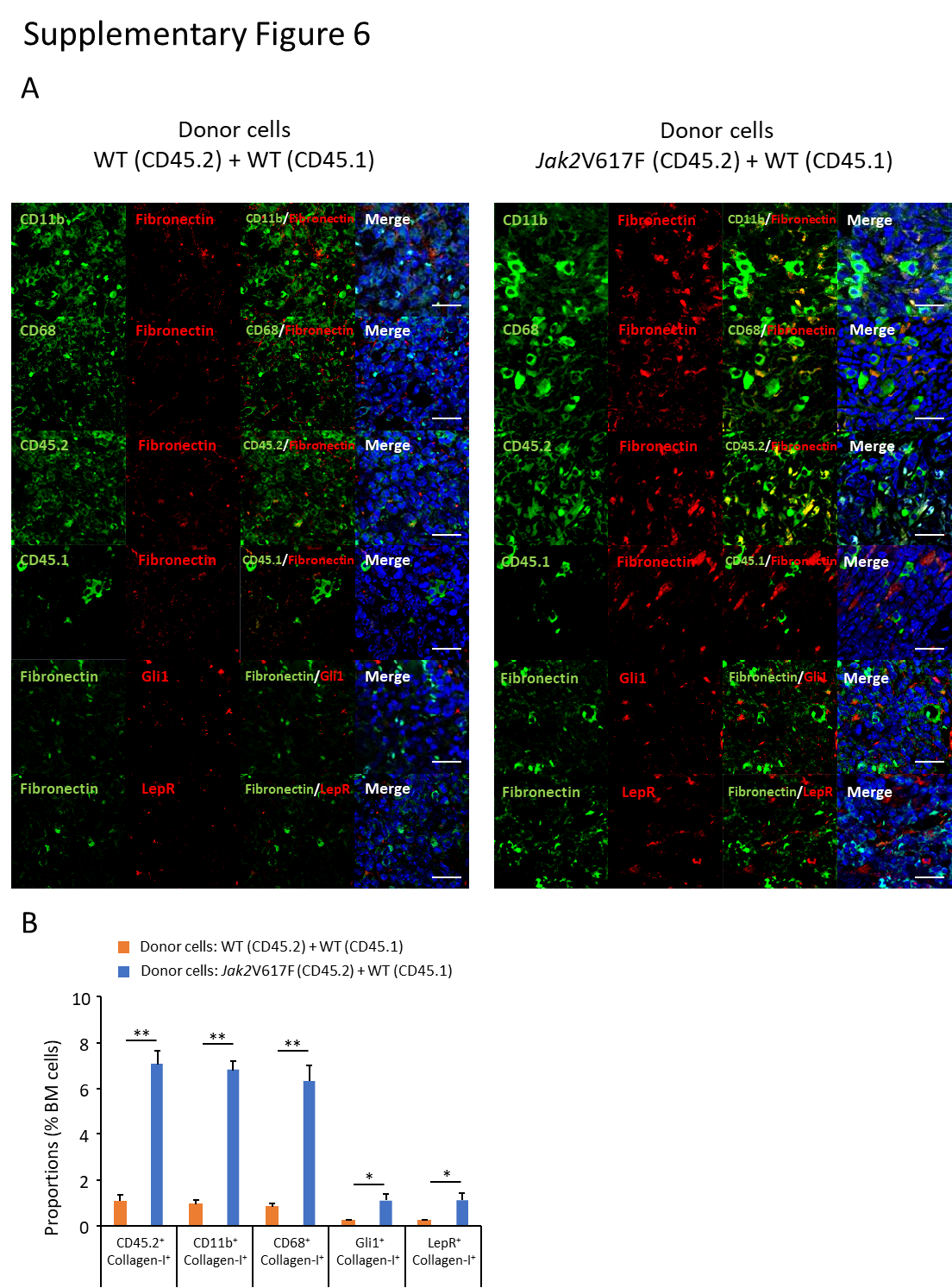
**

**
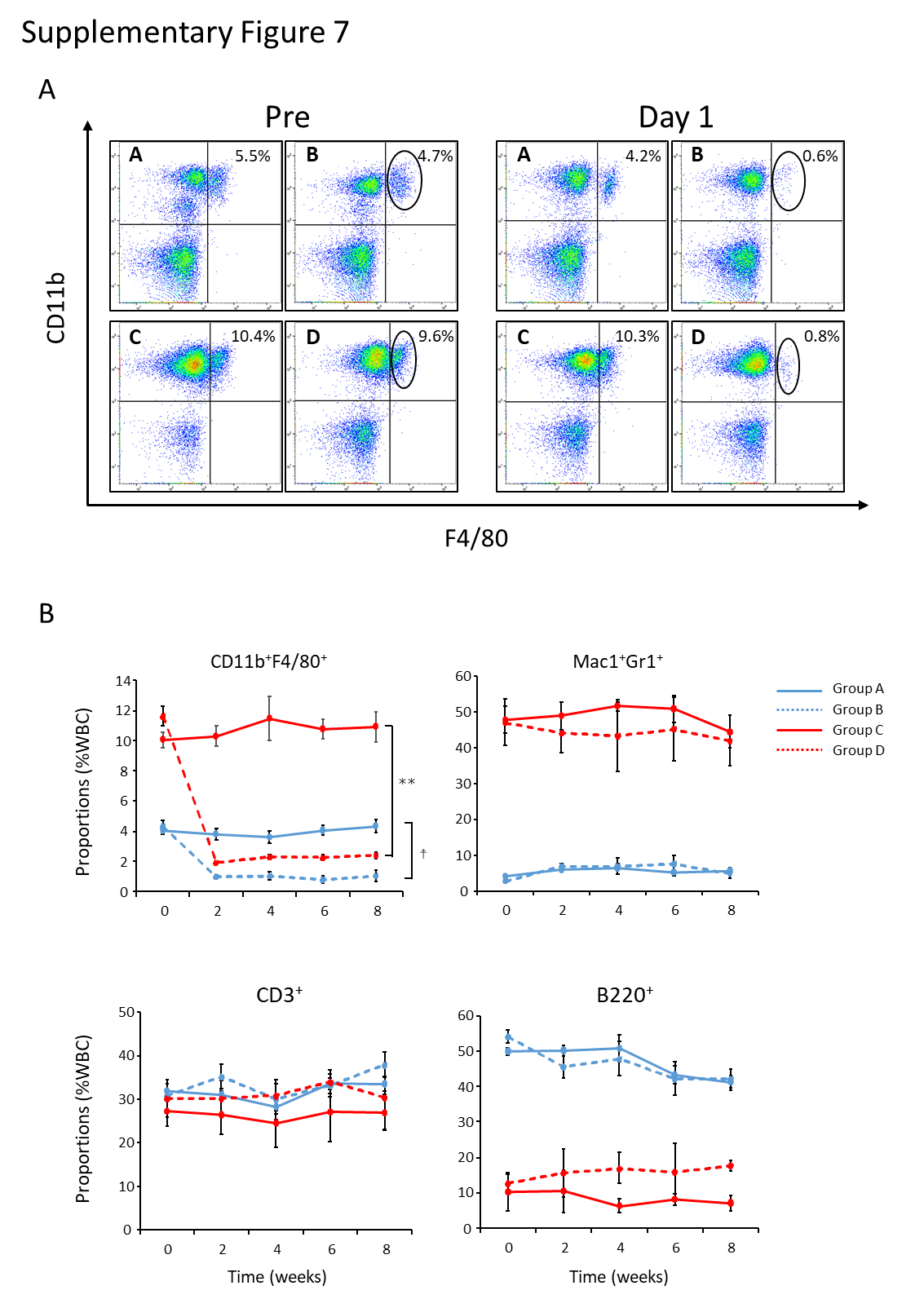
**

**
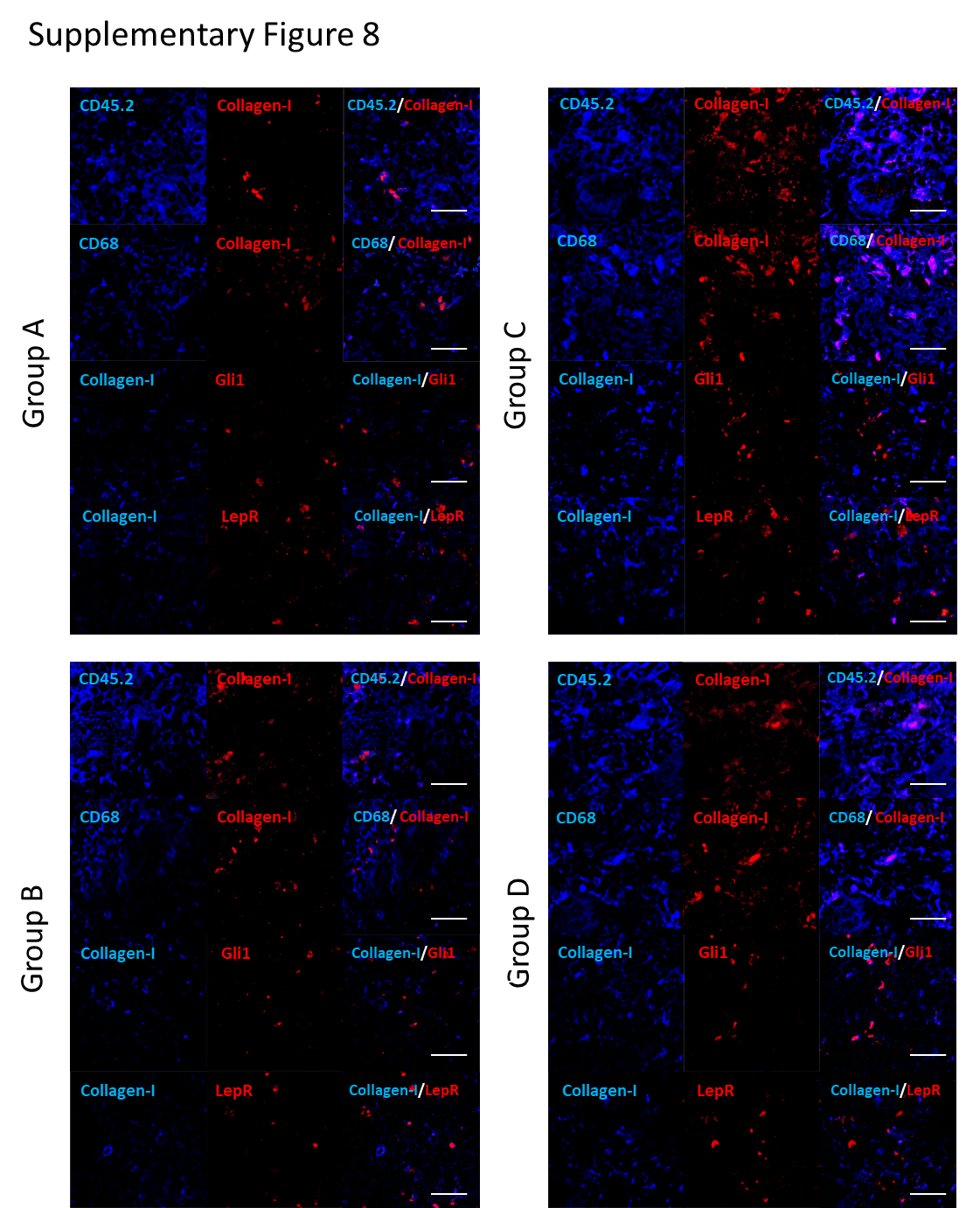
**

**
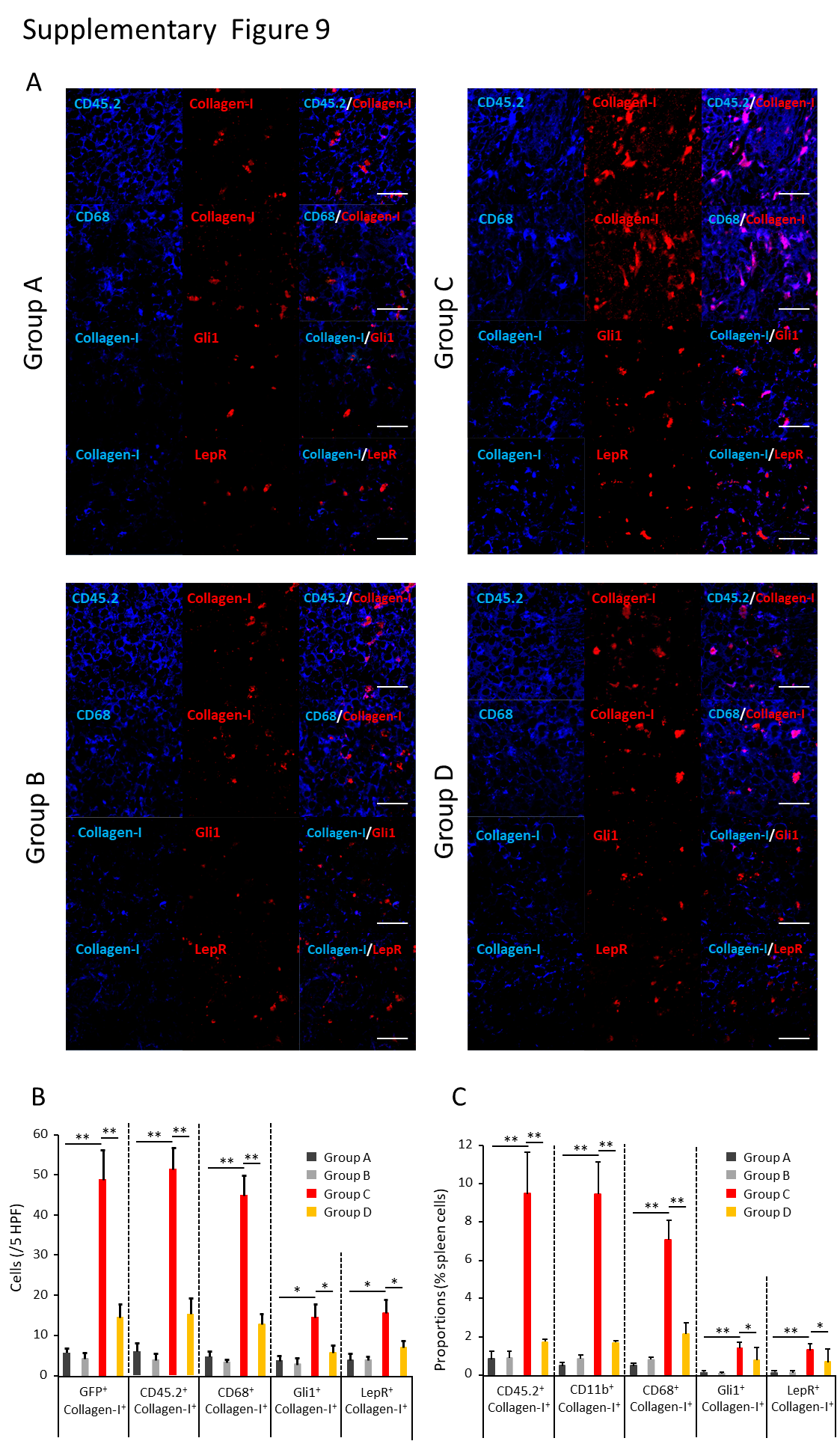
**

**
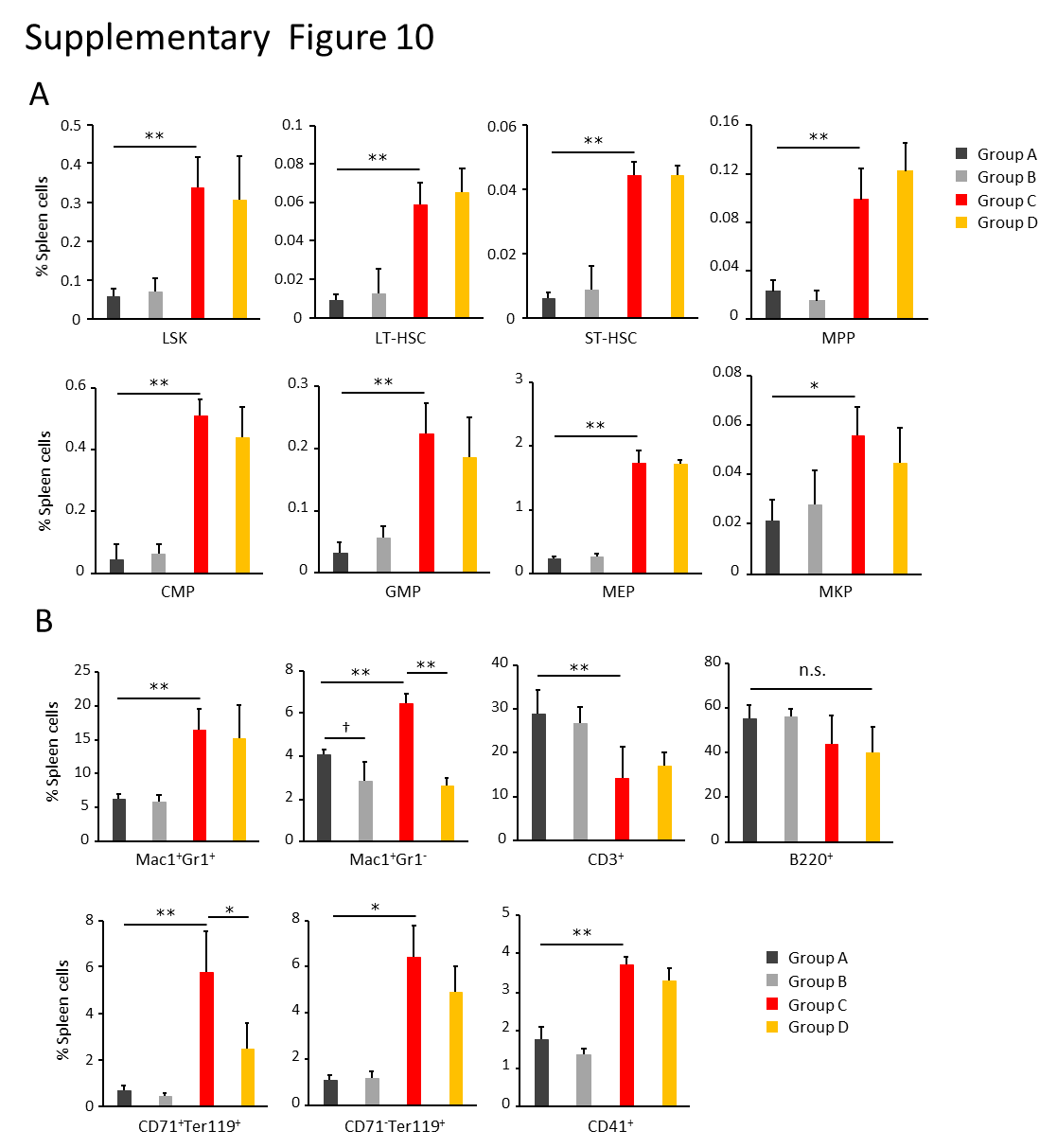
**

**
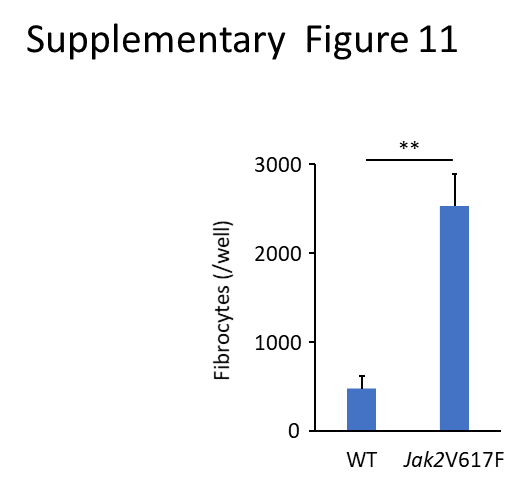
**

**
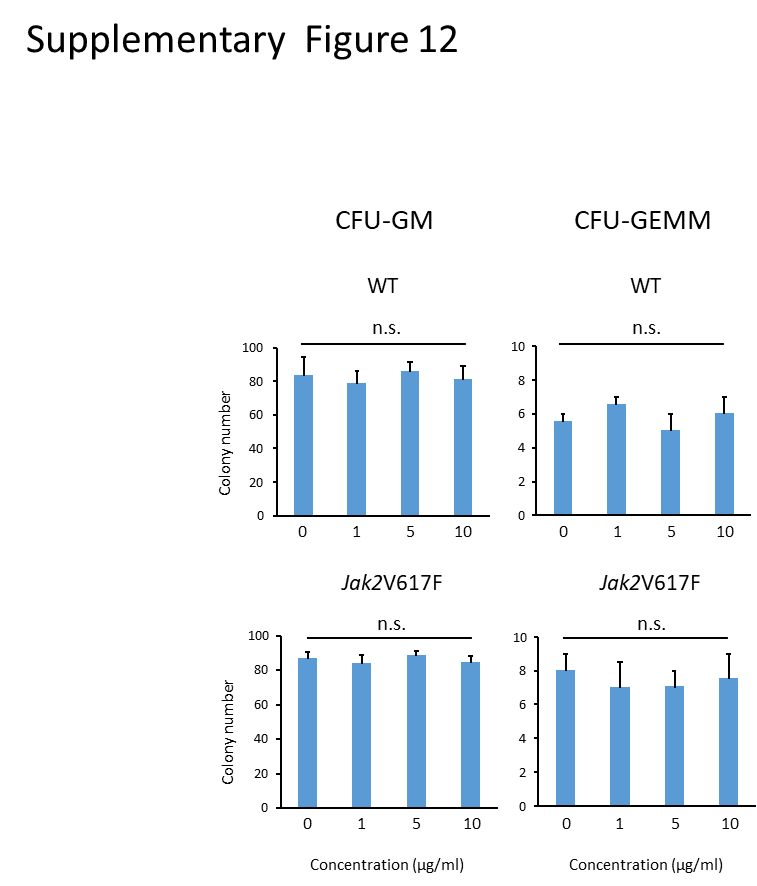
**

**Supplementary figure legends**

**Supplementary Figure 1. Increased number of collagen-producing neoplastic fibrocytes in BM of *Jak2*V617F TG mice**

Immunofluorescence imaging of BM sections from *Jak2*V617F TG (right) and WT mice (left). DAPI staining is shown in blue (merge). Bars: 20 μm. The representative result is shown in three independent experiments.

**Supplementary Figure 2. Collagen-producing myofibroblasts in BM of WT and *Jak2*V617F TG mice**

Immunofluorescence imaging of BM sections from *Jak2*V617F TG (right) and WT mice (left). DAPI staining is shown in blue (merge). Bars: 20 μm. The representative result is shown in three independent experiments.

**Supplementary Figure 3. Increased number of fibronectin-producing neoplastic fibrocytes in BM of *Jak2*V617F TG mice**

Immunofluorescence imaging of BM sections from *Jak2*V617F TG (right) and WT mice (left). DAPI staining is shown in blue (merge). Bars: 20 μm. The representative result is shown in three independent experiments.

**Supplementary Figure 4. Fibronectin-producing myofibroblasts in BM of WT and *Jak2*V617F TG mice**

Immunofluorescence imaging of BM sections from *Jak2*V617F TG (right) and WT mice (left). DAPI staining is shown in blue (merge). Bars: 20 μm. The representative result is shown in three independent experiments.

**Supplementary Figure 5. The number of neoplastic fibrocytes is increased in recipient mice transplanted with *Jak2*V617F FLCs**

(A) Hematoxylin and eosin (HE), reticulin silver, and Masson trichrome staining of BM sections from recipient mice transplanted with *Jak2*V617F FLCs and those transplanted with WT FLCs (n=3 in each group). Images from all six recipient mice are presented. Bars: 50 μm. (B) Representative immunofluorescence imaging of BM sections from recipient mice (CD45.1) transplanted with *Jak2*V617F FLCs (CD45.2) (right) and recipient mice (CD45.1) transplanted with WT FLCs (CD45.2) (left). DAPI staining is shown in blue (merge). Bars: 20 μm. The representative result is shown in three independent experiments. (C) The number of CD45.2^+^Collagen-I^+^, CD11b^+^Collagen-I^+^, and CD68^+^Collagen-I^+^ fibrocytes, as well as Gli1^+^Collagen-I^+^ and LepR^+^Collagen-I^+^ myofibroblasts in BM of recipient mice transplanted with *Jak2*V617F or WT FLCs, were determined by immunohistochemical analysis and are presented as the average of five random HPFs from three mice of each type. Data are expressed as means ± SEM. The 2-tailed student t-test was used. ***P* < 0.01. **P* < 0.05.

**Supplementary Figure 6. Detection of fibronectin-producing fibrocytes in BM from recipient mice transplanted with both *Jak2*V617F BMCs and WT BMCs.**

(A) Representative immunofluorescence imaging of BM sections from recipient mice (CD45.1) transplanted with both *Jak2*V617F BMCs (CD45.2) and WT BMCs (CD45.1) (right), and recipient mice (CD45.1) transplanted with both WT BMCs (CD45.2) and WT BMCs (CD45.1) (left) DAPI staining is shown in blue (merge). Bars: 20 μm. The representative result is shown in three independent experiments. (B) The proportion of CD45.2^+^Collagen-I^+^, CD11b^+^Collagen-I^+^, and CD68^+^Collagen-I^+^ fibrocytes and Gli1^+^Collagen-I^+^ and LepR^+^Collagen-I^+^ myofibroblasts in BMCs of recipient mice (CD45.1) transplanted with the mixture of *Jak2*V617F BMCs (CD45.2) plus WT BMCs (CD45.1), and recipient mice (CD45.1) transplanted with the mixture of WT BMCs (CD45.2) plus WT BMCs (CD45.1) by FACS analysis (n=5 in each group). Data are expressed as means ± SEM. The 2-tailed student t-test was used. ***P* < 0.01. **P* < 0.05.

**Supplementary Figure 7. Changes in PB leukocyte differentiation during DT treatment**

(A) Representative plots of FACS analysis of CD11b^+^F4/80^+^ monocytes. The reproducibility was confirmed using eight mice in each group. (B) CD11b^+^F4/80^+^ monocyte depletion is maintained during 8-week DT treatment (n=12 in group A and C, n=14 in group B and D). Data are expressed as means ± SEM. ANOVA with repeated measures was used. ***P* < 0.01. ^☨^*P* < 0.05.

**Supplementary Figure 8. Monocyte depletion reduces collagen-producing fibrocytes in BM from recipient mice transplanted with *Jak2*V617F BMCs.**

Immunofluorescence imaging of BM sections from recipient mice transplanted with *Jak2*V617F/CD11b-DTR BMCs or CD11b-DTR BMCs. Bars: 20 μm. The representative result is shown in three independent experiments.

**Supplementary Figure 9. Monocyte depletion reduces collagen-producing fibrocytes in spleens of recipient mice transplanted with *Jak2*V617F BMCs.**

(A) Immunofluorescence imaging of spleen sections from recipient mice transplanted with *Jak2*V617F/CD11b-DTR BMCs or CD11b-DTR BMCs. Bars: 20 μm. The representative result is shown in three independent experiments.

(B) Quantification of splenic GFP^+^Collagen-I^+^, CD45.2^+^Collagen-I^+^, and CD68^+^Collagen-I^+^ fibrocytes, as well as Gli1^+^Collagen-I^+^ and LepR^+^Collagen-I^+^ myofibroblasts, are presented as the average of five random HPFs from three mice of each type. (C) FACS analysis showing the proportion of fibrocytes and myofibroblasts in the spleens of recipient mice transplanted with CD11b-DTR BMCs or *Jak2*V617F/CD11b-DTR BMCs (n=6 in group A and B, n=8 in group C and D). Data are expressed as means ± SEM. One-way ANOVA followed by the Tukey-Kramer test was used (B, C). ***P* < 0.01. **P* < 0.05.

**Supplementary Figure 10. The effect of monocyte depletion on spleen progenitor cells.**

(A) The proportion of LSKs, long-term HSCs, short-term HSCs, MPPs, CMPs, GMPs, MEPs, and MKPs in spleens was analyzed by flow cytometry (n=6 in group A and B, n=8 in group C and D). (B) Analysis of Mac1^+^Gr1^+^ granulocytes, Mac1^+^Gr1^-^ monocytes, CD3^+^ T cells, B220^+^ B cells, CD71^+^Ter119^+^ early erythroblasts, CD71^-^Ter119^+^ late erythroblasts, and CD41^+^ megakaryocytes from the spleens of recipient mice transplanted with *Jak2*V617F/CD11b-DTR BMCs or CD11b-DTR BMCs were analyzed by flow cytometry (n=6 in group A and B, n=8 in group C and D). Data are expressed as means ± SEM. One-way ANOVA followed by the Tukey-Kramer test was used (A, B). ***P* < 0.01. **P* < 0.05. ^☨^*P* < 0.05. n.s.: not significant.

**Supplementary Figure 11.** **The number of fibrocytes per well after 5-day culture.** Fibrocytes were enumerated by morphology. Data are expressed as means ± SEM. The 2-tailed student t-test was used. ***P* < 0.01. The reproducibility was confirmed by two experiments.

**Supplementary Figure 12. TGF-β1-neutralizing antibodies had little effect on BM colony formation.**

TGF-β1-neutralizing antibodies or isotype control were added in BM colony formation assay. Data are expressed as means ± SEM. One-way ANOVA followed by the Tukey-Kramer test was used. n.s.: not significant. The reproducibility was confirmed by two experiments.
